# Asymmetric PTEN Distribution Regulated by Spatial Heterogeneity in Membrane-Binding State Transitions

**DOI:** 10.1371/journal.pcbi.1002862

**Published:** 2013-01-10

**Authors:** Satomi Matsuoka, Tatsuo Shibata, Masahiro Ueda

**Affiliations:** 1 Laboratory for Cell Signaling Dynamics, RIKEN Quantitative Biology Center, Suita, Japan; 2 Laboratories for Nanobiology, Graduate School of Frontier Biosciences, Osaka University, Suita, Japan; 3 CREST, Japan Science and Technology Agency (JST), Suita, Japan; 4 Laboratories for Physical Biology, RIKEN Center for Developmental Biology, Kobe, Japan; 5 Laboratory of Single Molecule Biology, Department of Biological Sciences, Graduate School of Science, Osaka University, Toyonaka, Japan; University of Notre Dame, United States of America

## Abstract

The molecular mechanisms that underlie asymmetric PTEN distribution at the posterior of polarized motile cells and regulate anterior pseudopod formation were addressed by novel single-molecule tracking analysis. Heterogeneity in the lateral mobility of PTEN on a membrane indicated the existence of three membrane-binding states with different diffusion coefficients and membrane-binding lifetimes. The stochastic state transition kinetics of PTEN among these three states were suggested to be regulated spatially along the cell polarity such that only the stable binding state is selectively suppressed at the anterior membrane to cause local PTEN depletion. By incorporating experimentally observed kinetic parameters into a simple mathematical model, the asymmetric PTEN distribution can be explained quantitatively to illustrate the regulatory mechanisms for cellular asymmetry based on an essential causal link between individual stochastic reactions and stable localizations of the ensemble.

## Author Summary

Signaling molecules can adopt multiple states that are characterized by intermolecular interactions and chemical modifications, and can thus describe a mechanistic basis for signal transduction. To understand the molecular mechanisms driving signaling reactions, the kinetics and spatial regulation of the state transitions are indispensable. However, using data from single-molecule imaging observations, state transition behaviors of only signaling molecules integrated into the plasma membrane have been analyzed. Here, we propose a novel analysis method which estimates the kinetics of the state transitions and membrane dissociation of signaling molecules that shuttle between the cytoplasm and membrane, a method based on the statistics of the diffusive mobilities of individual molecules. Applying the method to single-molecule tracking analysis of PTEN, a key player in the cellular polarity of *Dictyostelium discoideum* and mammalian neutrophils, we demonstrate the validity of our method for investigating the mechanism of asymmetric membrane localization. The method enables a quantitative prediction of signaling activities at local membrane regions and therefore provides a powerful tool for understanding the essential mechanisms of a signaling system.

## Introduction

Intracellular signal transduction at the cell membrane mediates various extracellular signals inside the cell for proper environmental adaptation. Signaling molecules achieve their function in large part by translocating between the membrane and cytoplasm to mediate a variety of signaling systems including Raf, PKB and PLC in growth factor signaling, Zap-70 in T-cell receptor signaling, Rap1 in integrin signaling and heterotrimeric G protein, Crac and PTEN in *Dictyostelium* chemotactic signaling [1], [2], [3], [4], [5], [6]. The intracellular distribution of signaling molecules is regulated dynamically via their transient and repetitive associations with the membrane, a phenomenon that can be viewed as dynamic shuttling between the membrane and cytoplasm. Upon environmental changes, the shuttling is modulated spatially and temporally in response to the multiple membrane-binding states that arise with changes in a signaling molecule's interactions with other molecules such as membrane receptors, effectors, and lipids. Spatial and temporal heterogeneities in the molecular states, the shuttling itself and finally the number of molecules interacting with the membrane arise inevitably during the signal transduction processes, which then act as a basis for cellular responses. Thus, signal transduction can be regarded as a molecular process that regulates the state transition. Nevertheless, few studies have described membrane localization based on the molecular reactions of membrane associations and dissociations or state transitions.

One reason is the difficulty of directly observing and measuring signaling reactions on the membranes of living cells, although this has been growing increasingly more feasible with gains in single-molecule imaging techniques. For example, using total internal reflection fluorescence microscopy (TIRFM), we have been able to trace the behavior of a single molecule while it is bound to a cell membrane [7], [8], [9], [10], allowing us to follow a series of signaling reactions while the signaling molecules associate with, move laterally along and dissociate from the membrane. The membrane-binding state can be characterized by the lateral mobility of diffusion and/or membrane-binding lifetimes, which are quantified by statistically describing the single-molecule trajectories. For example, diffusion analysis considers the spatial distance between two positions of a molecule over a unit time interval of the trajectory [11], [12], [13], [14]. In contrast, lifetime analysis considers the time duration of individual trajectories from the onset to completion of a membrane association [15], [16]. Since these two analyses focus exclusively on the spatial or temporal aspects of single-molecular behavior, respectively, heterogeneity in one cannot be easily correlated with the other, which complicates our understanding of the details of the state transition kinetics and the relevant signal transduction mechanisms. An ideal analysis method, therefore, will unify the spatial and temporal information of single-molecule trajectories.

In the present study, we propose a novel and general statistical method for the single-molecule tracking analysis of signaling molecules on the membrane of living cells, which we name lifetime-diffusion analysis. The method estimates state transition kinetics and membrane-binding lifetimes from single-molecule trajectories by correlating each membrane-binding state to the characteristic lateral mobility. The method is here shown valid for the PtdIns(3,4,5)P_3_ phosphatase PTEN (a phosphatase and tensin homologue deleted on chromosome 10). PTEN, which was first identified as a tumor suppressor in mammalian cells, is involved in chemotactic signaling in *Dictyostelium discoideum* [6], [17]. It has been observed that PTEN is excluded from the anterior membrane of polarized *Dictyostelium* cells that undergo chemotaxis in response to a chemical gradient and that a PtdIns(3,4,5)P_3_-enriched domain arises at the cell end with the higher gradient concentration [18], [19]. The domain is generated in an ultrasensitive and self-organizing manner and serves as a signal to activate pseudopod formation concerting with other signals in parallel signaling pathways, with the posterior localization of PTEN being critical for the anterior confinement of the patch and efficient directed migration [6], [19], [20], [21], [22], [23]. Various mathematical models have been proposed to understand the underlying mechanism for the domain formation assuming essential molecular reactions and movements involving catalytic processes by PI3K and PTEN, lateral diffusion of PtdIns lipids and interactions between the enzymes and lipid molecules [24], [25], [26]. To discriminate the correct model, we require information on local differences in the reactions and movements, as these explain the global domain generation, and also need to observe and analyze the behaviors of PtdIns lipids, PI3K and PTEN on the membrane of living cells according to the structures of the molecular ensemble. By using our lifetime–diffusion analysis, we here investigated the molecular mechanism driving asymmetric PTEN distribution in migrating *Dictyostelium* cells. A kinetic model was obtained and suggests that PTEN molecules exhibit stochastic transitions among three states with different diffusion coefficients and membrane-binding lifetimes. The asymmetric distributions were explained quantitatively by the spatially regulated heterogeneity of the state transition kinetics, illustrating the regulatory mechanisms of PTEN distribution with single-molecular resolution. Our lifetime-diffusion analysis described here can be applied in general to other membrane-bound signaling molecules on the membrane.

## Results

### Heterogeneity in PTEN membrane-binding properties

**Figure 1:**
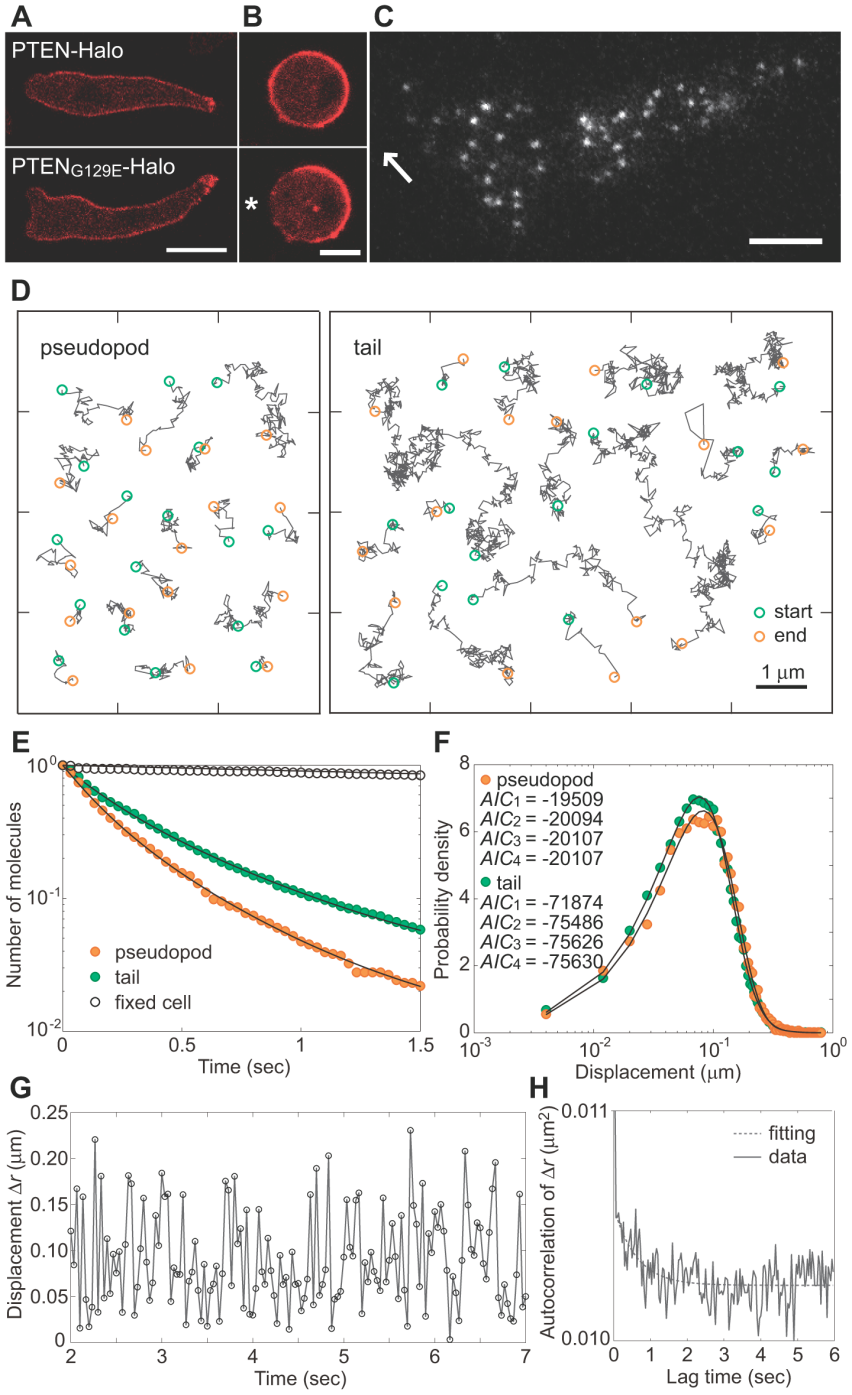
Heterogeneities in PTEN molecules on the membrane of polarized cells. (A) Fluorescent images of *Dictyostelium discoideum* cells expressing PTEN-Halo (*top*) and PTEN_G129E_-Halo (*bottom*) labeled with TMR-conjugated HaloTag ligand. Cells moved leftward. Scale bar, 5 µm. (B) Asymmetric distribution of PTEN_G129E_-Halo on the membrane upon stimulation with a cAMP gradient. The cell was treated with 5 µM Latrunculin A (*top*). The asterisk indicates the position of the pipette tip containing 1 µM cAMP (*bottom*). Scale bar, 5 µm. (C) Single molecules of PTEN_G129E_ bound to the membrane of a migrating cell. The arrow indicates the direction of movement. Scale bar, 5 µm. (D) Typical trajectories of single PTEN_G129E_ molecules observed at the pseudopod (*left*) and tail (*right*). (E) Dissociation curves of PTEN_G129E_ molecules observed at the pseudopod and tail. Fitting curves are from Eq. S11 using the parameter values described in Table 1. (F) Distributions of displacement during 33 ms of observation at the pseudopod and tail. Fitting curves are from Eq.11 assuming a three-state model. Diffusion coefficients and their proportions are described in Table 1 and Fig. 5C. (G) The time series of displacement of a PTEN_G129E_ molecule over a 33 ms window (excerpted from the whole trajectory observed at the tail). (H) Autocorrelation function calculated from the time series of displacements for 10 molecules observed at the tail. The fitting function is *y* = a*exp(−*Kt*)+*b* with *K* = 1.64 s^−1^. See also Movie S1.

In order to understand the molecular mechanisms of PTEN asymmetric localization, we first examined the intracellular localization of the mutant PTEN_G129E_, which has a G129E substitution that leads to no substrate binding or phosphatase activity against PtdIns(3,4,5)P_3_ [27], [28]. PTEN_G129E_ was found localized on the membrane except in the pseudopod, which is similar to wild-type PTEN properties (Figure 1A). Asymmetric PTEN_G129E_ distribution on the membrane was induced by applying concentration gradients of the chemoattractant cAMP to cells lacking functional actin cytoskeletons, which resulted in stimulation-induced local depletion of PTEN_G129E_ (Figure 1B). Therefore, neither PtdIns(3,4,5)P_3_ binding nor enzymatic activity is a prerequisite for the asymmetric distribution of PTEN on the membrane of *Dictyostelium* cells that respond to cAMP gradients. Thus, because these properties simplify the analysis of kinetics before membrane dissociation, we used PTEN_G129E_ for the subsequent analysis.

Single molecules of PTEN_G129E_-Halo conjugated with tetramethylrhodamine (TMR) were visualized under TIRFM (Figure 1C and Movie S1). Visualized fluorescent spots showed quantized photobleaching, fluorescence intensities and spot sizes typical of single fluorophores (Figures S1A, see Text S1 for detail). The region of the membrane corresponding to the pseudopod could be clearly distinguished from the rest of the membrane, as the number of PTEN_G129E_ molecules there was minimal (Figure 1C). The trajectories of single PTEN_G129E_ molecules showed different behaviors in time and space between the pseudopod and elsewhere (Figure 1D). Membrane dissociation occurred faster on the membrane at the pseudopod than at the tail (Figure 1E). The dissociation curves had decay rates faster than that of photobleaching, indicating that PTEN_G129E_ molecules shuttle between the membrane and cytoplasm. The photobleaching rate constant was 0.1 s^−1^ when immobilized PTEN_G129E_ molecules on a membrane of fixed cells were visualized. The lateral diffusion was found to be slightly faster in the pseudopod than in the tail (Figure 1F). Furthermore, mean square displacement (MSD) increased linearly with time, suggesting that individual molecules showed normal diffusion, not confined diffusion due to a compartmentalization (see, for example, [29]) or super diffusion due to a directional motility (see, for example, [12]) (Figure S1B). Therefore, on average, PTEN_G129E_ molecules exhibited faster membrane dissociation and faster lateral diffusion at the pseudopod than at the rest of the polarized cell. In addition, the temporal correlation of the diffusion mobility was examined by using a time series of the displacements made from each trajectory (Figure 1G). The autocorrelation function exhibits an exponential decay with a rate constant of 1.64 s^−1^, indicating an alternation in the diffusion mobility (Figure 1H) [13].

### Three fundamental models describing lateral diffusion, state transitions and membrane dissociation

**Figure 2:**
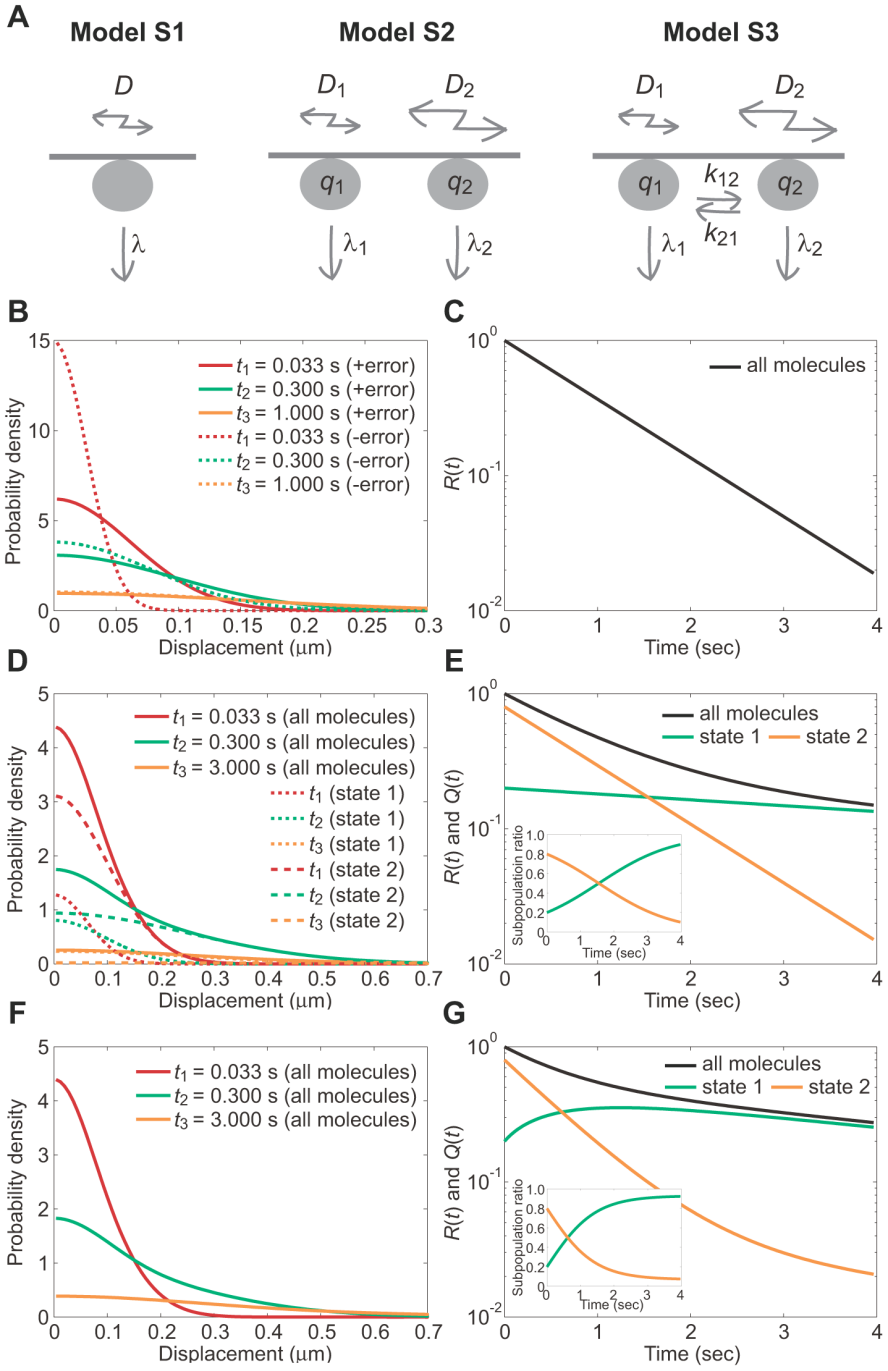
Models of membrane-bound signaling molecules exhibiting diffusion, state transitions and membrane dissociation. (A) Schematic view of the three principle models. (B,D,F) The probability density function (PDF) of molecular position at *t* = 0.033, 0.3 and 1 (B) or 0.033, 0.3 and 3 (D,F). (C,E,G) The membrane residence probability, *R*(*t*) (*black*), and the subpopulation probability, *Q*(*t*), for state 1 (*green*) and state 2 (*orange*). (insets in E and G) Time series of the subpopulation ratios. (B,C) Model S1. The PDFs before (*dotted lines*) and after (*solid lines*) incorporating the measurement error are shown. *D* = 0.01, *λ* = 1.00 and *ε* = 0.04. (D,E) Model S2. *D*_1_ = 0.01, *D*_2_ = 0.10, *λ*_1_ = 0.10, *λ*_2_ = 1.00, *q*_1_ = 0.20 and *ε* = 0.04. (F,G) Model S3. *D*_1_ = 0.01, *D*_2_ = 0.10, *λ*_1_ = 0.10, *λ*_2_ = 1.00, *k*_12_ = 0.10, *k*_21_ = 0.50, *q*_1_ = 0.20 and *ε* = 0.04. *D*, µm^2^s^−1^; *λ*, *k*, s^−1^; *ε*, µm. PDFs for Models S2 and S3 incorporate the measurement error. See also Figures S1.

To identify the possible multiple states that a membrane-bound molecule adopts and to characterize the corresponding state transition kinetics, we developed a novel single-molecule tracking analysis method, lifetime-diffusion analysis. We previously reported a method which can be used for analyzing the multistate kinetics of membrane-integrated molecules based on the simplest of three theoretical models [13], which are only applicable to molecules like receptors, channels and adhesion proteins that are incorporated into the membrane lipid bilayer. Here, we extend these models to cases when molecules are shuttling between the membrane and cytoplasm (Models S1–S3 in Figure 2A). The models are appealing, because they describe the essential behaviors of signaling molecules that show simple diffusion along a membrane, and therefore provide a theoretical basis for the analysis method. Should a molecule show confined or super diffusion, our method can still be applied but requires amendments to the diffusion equations to account.

#### Simple diffusion in the presence of membrane dissociation (Model S1)

Consider a molecule that exhibits transient membrane associations and normal diffusion on the membrane plane (Figure 2A, Model S1). Dissociation occurs independently of diffusion, so that a probability density function (PDF) of the position of the molecule, *P*(*x*,*y*,*t*), satisfies the diffusion equation,
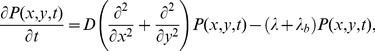
(1)where *D* and *λ* represent the diffusion coefficient and the dissociation rate constant of the molecule, respectively. *λ*_b_ is the rate constant of photobleaching, which occurs on the conjugated fluorophore independent of membrane dissociation. *t* represents the time after the onset of membrane binding. Assuming that the molecule is located at the origin when it first appears on the membrane, the PDF can be written as,
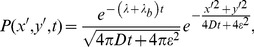
(2)where (*x*′, *y*′) represents the position of the molecule as measured from the single-molecule images, and therefore contains errors with a standard deviation (SD) of *ε* from the genuine position, (*x*, *y*) [13]. Here we assume no spatial anisotropy in the membrane, and describe the PDFs and their plots one-dimensionally for convenience (Figure 2B). Over time, the distribution becomes wider while the mean is constant at the origin and the error becomes less significant. At the same time, the area below the curve decreases, corresponding to a decrease in the probability that the molecule remains bound to the membrane. As shown in Figure 2C, the membrane residence probability after the onset of membrane-binding is given by the integral of Eq. 2 with respect to (*x*′, *y*′) as,
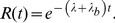
(3)After an infinite time, the PDF approaches 0 irrespective of position. When derived theoretically from an appropriate model, *R*(*t*) can explain the dissociation curve obtained experimentally. The actual rate constant, *λ*, is estimated by subtracting *λ*_b_ from the measured rate constant of the fluorescent spot disappearance, *λ*′ = *λ*+*λ*_b_.

#### Two states with different diffusion coefficients and dissociation rate constants (Model S2)

Consider a molecule that has two possible states on the membrane. At first, no transitions are assumed and the molecule adopts either state throughout the membrane association (Figure 2A, Model S2). Each state has a characteristic diffusion coefficient and dissociation rate constant. Letting these be *D*_1_ and *λ*_1_ for state 1 and *D*_2_ and *λ*_2_ for state 2, the PDFs of the molecule position in state 1 and 2 follows,
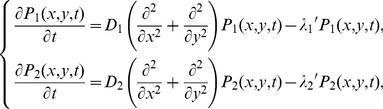
(4)where *λ*_1_′ = *λ*_1_+*λ*_b_ and *λ*_2_′ = *λ*_2_+*λ*_b_. Which state the molecule adopts is determined when it first associates with the membrane. Given that the initial probability is *q*_1_ and *q*_2_ for state 1 and 2, respectively, where *q*_1_+*q*_2_ = 1, the PDF describing the ensemble of the molecule comprises two PDFs describing states 1 and 2 (Figure 2D, Eq. S3). The integral of the PDF with respect to *x* and *y* gives rise to the membrane residence probability, *R*(*t*), shown in Figure 2E as,
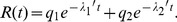
(5)We focus on the subpopulation of molecules that take states 1 or 2 at time *t*. The subpopulation probabilities, *Q*_1_(*t*) and *Q*_2_(*t*), for states 1 and 2, respectively, decay monotonically due to membrane dissociation with the respective rate constants, 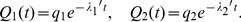
(6)When the ratio of one subpopulation to the whole is taken, it temporally varies from the initial value (Figure 2E, inset). *Q*_1_(*t*)/*R*(*t*) changes from *q*_1_ to 1 as *t* increases assuming *λ*_1_′≪*λ*_2_′ (Figures 2D and 2E, inset).

#### Two-state model in the presence of state transitions (Model S3)

Consider a molecule that exhibits a state transition between two states during membrane association (Figure 2A, Model S3). The PDFs of the molecule adopting states 1 or 2 follows the diffusion equations,
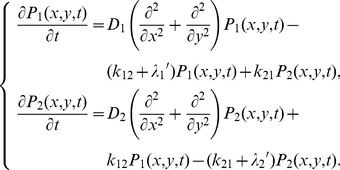
(7)It should be noted that the transition rate constants, *k*_12_ and *k*_21_, are independent of the photobleaching rate constant, *λ*_b_. The diffusion equations can be solved after taking the Fourier transform, with the inverse transformation being performed by numerical integration to obtain the PDFs (Figure 2F, see Text S1 for details). The membrane residence probability *R*(*t*) is written as,
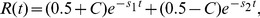
(8)and the subpopulation probabilities *Q*_1_(*t*) and *Q*_2_(*t*) with *R*(*t*) = *Q*_1_(*t*)+*Q*_2_(*t*) are given by
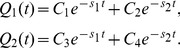
(9)Details of the constants, *C* and *C*_N_, are described in Text S1. Although the membrane residence probability follows a sum of two exponential functions (Figure 2G), the exponential decay rates do not equal the membrane dissociation rates of the two states (Text S1). The subpopulation probabilities exhibit biphasic decays because of dissociation and the transitions from or to the other state. Individual molecules can show repeated transitions between two states over time after the onset of membrane association. After a sufficiently long time, stationary behaviors are such that the ratio of one subpopulation to the whole is approximately constant (Figure 2G, inset), meaning that the molecules reach a steady state with respect to the state transition. Consistently, the decay profiles of each subpopulation eventually begin to exhibit the same rate, which was then used to test for the presence of state transitions, as described below.

### Method to apply lifetime-diffusion analysis to single molecule trajectories

**Figure 3:**
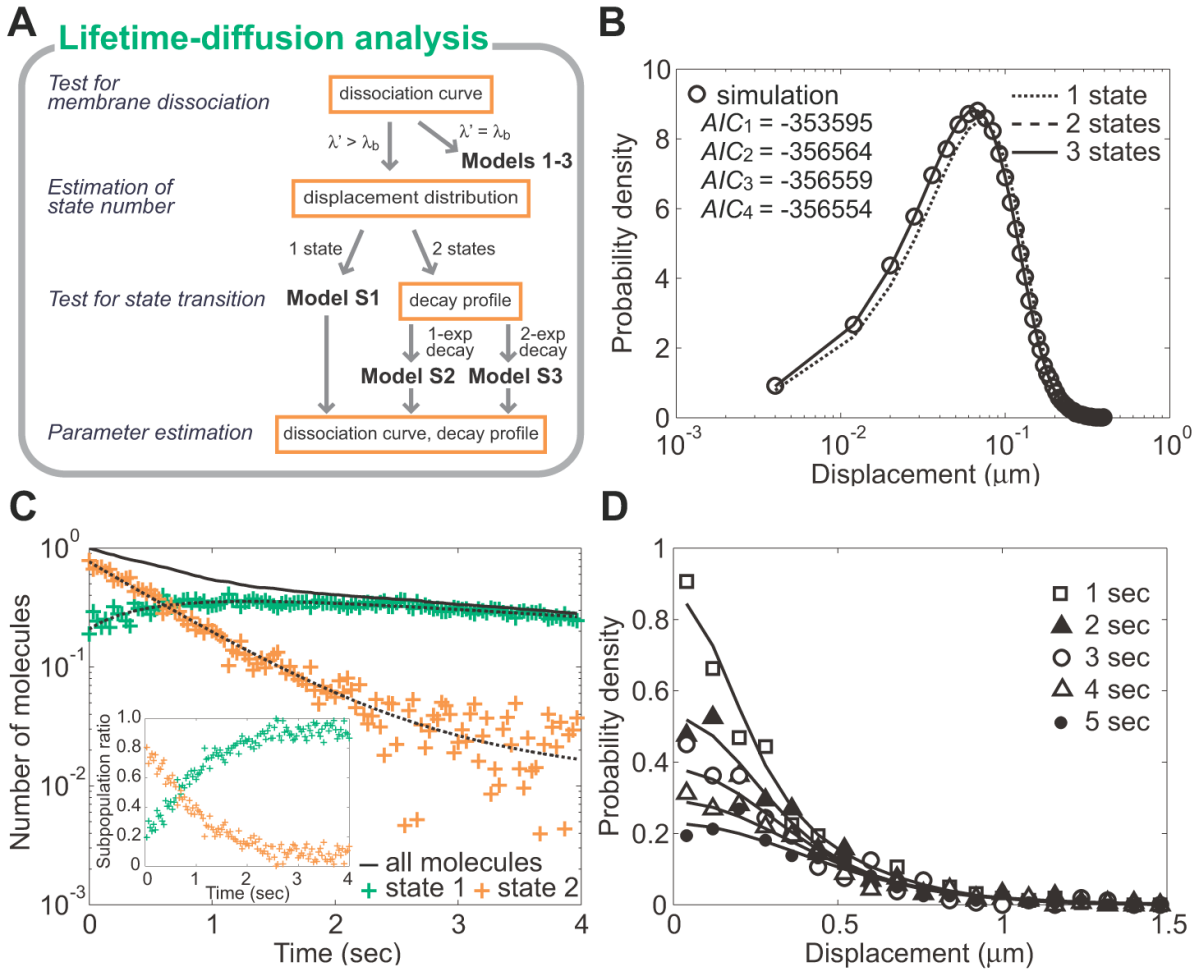
Lifetime-diffusion analysis. (A) An overview of lifetime-diffusion analysis. (B) Estimation of the state number. The trajectories generated by a numerical simulation using the same model as in Figure 2F are analyzed below. Displacement during a 33 ms window (*open circle*) was used for MLE (*lines*), which give AIC values (*inset*) that suggest the state number is two. The estimated diffusion coefficients are *D*_1_ = 0.011 and *D*_2_ = 0.100 µm^2^s^−1^. (C) Test for the state transitions and parameter estimation. The decay profiles of the subpopulations with diffusion coefficients *D*_1_ (*green*) and *D*_2_ (*orange*) show that the molecules exhibit state transitions. The estimated parameters are *λ*_1_ = 0.110, *λ*_2_ = 0.940, *k*_12_ = 0.068, *k*_21_ = 0.474 and *q*_1_ = 0.197. (inset) Time series of the subpopulation ratios. (D) Consistency between the obtained model and the data. Histograms of molecular position are shown at five time points: *t*_1_ = 1, *t*_2_ = 2, *t*_3_ = 3, *t*_4_ = 4 and *t*_5_ = 5 s. See also Figure S2.

In order to clarify the multistate kinetics of a molecule, lifetime-diffusion analysis (Figure 3A), which consists of the following series of steps, is done. First, we determine whether the molecule exhibits membrane dissociation or not. Second, we count the number of membrane-binding states with different diffusion coefficients. Third, we observe whether the molecule exhibits state transitions. Finally, we construct a model consistent with the experimental results and estimate the kinetic parameters by fitting the data to theoretical functions derived from the model.

In the first step, membrane dissociation is examined by comparing the disappearance rate of single-molecule fluorescence with the photobleaching rate of the fluorophore itself (Figure 1E). When the fluorophore is conjugated to membrane-integrated molecules or immobilized onto a glass surface, the fluorescence will disappear with the photobleaching rate. When it is conjugated to molecules shuttling between the membrane and cytoplasm, however, the disappearance rate becomes faster than the photobleaching rate due to the additional membrane dissociation rate. The number of fluorophores undergoing photobleaching will decay with time as follows,
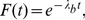
(10)where *λ_b_* is the photobleaching rate constant and can be assumed constant throughout the analysis when excitation conditions are unchanged (Text S1).

In the second step, the number of states with different diffusion coefficients is determined by statistical analysis of the displacements [13]. In the trajectories, displacement, Δ*r*(*t*) = ((*x*′(*t*+Δ*t*)−*x*′(*t*))^2^+(*y*′(*t*+Δ*t*)−*y*′(*t*))^2^)^1/2^, is calculated at an arbitrary *t* with a unit time interval Δ*t*. Under ideal conditions where the molecules adopt one of multiple membrane-binding states and do not change their state during Δ*t*, the displacement distribution can be regarded as a mixture of distributions with different diffusion coefficients and described by the PDF,
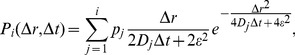
(11)where 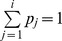. *i* indicates the state number, and *D_j_* and *p_j_* represent the diffusion coefficient of the *j*-th state and its proportion relative to all states, respectively. *ε* is the SD of the position error of the fluorescence spots. The minimum state number is defined as that when the theoretical PDF can fit well the experimental distribution and is determined by using Akaike Information Criterion (AIC) values calculated after the maximum likelihood estimation (MLE) for each state number [13], [30] (see Text S1 for details). Figure 3B shows a displacement distribution obtained from 1000 trajectories generated by numerical simulations using Model S3. Two or three states were sufficient to fit the distribution to Eq. 11 well. Based on the *AIC*_2_ value, we concluded that the molecule adopts two states. The estimated diffusion coefficients were *D*_1_ = 0.011 and *D*_2_ = 0.100 µm^2^s^−1^, which are almost the same as those used for the numerical simulation of the trajectories. For an accurate estimate of the diffusion coefficient, Eq. 11 should incorporate an *ε* value that is quantified prior to the MLE, which can be done by fitting *MSD*(Δ*t*) = 4D^*^Δ*t*+4*ε* ^2^ to the calculated MSD (Figure S1B, inset, Table 1). Assuming a mean diffusion coefficient D^*^ = 0, the *MSD*(Δ*t*) of immobilized molecules corresponds to 4*ε* ^2^ (see [13] for methods).

**Table 1:** Estimated parameters (three-state model).

	PTEN_G129E_						PTEN_G129E;Δ15_	
	No polarity		Posterior		Anterior		No polarity	
**Parameters**		CI#		CI#		CI#		CI#
*D*_1_ (µm^2^s^−1^)	0.035	0.026	0.034	0.028	0.039	−0.015	0.013	0.004
		0.045		0.040		0.093		0.023
*D*_2_ (µm^2^s^−1^)	0.133	0.109	0.150	0.117	0.115	0.048	0.161	0.131
		0.156		0.183		0.181		0.192
*D*_3_ (µm^2^s^−1^)	0.693	0.589	0.722	0.579	0.557	0.349	0.861	0.729
		0.796		0.866		0.764		0.994
*p*_1_	0.479		0.639		0.342		0.361	
*p*_2_	0.480		0.331		0.612		0.526	
*p*_3_	0.041		0.030		0.046		0.113	
*q*_1_	0.238		0.298		0.041		0.180	
*q*_2_	0.606		0.581		0.851		0.561	
*q*_3_	0.157		0.121		0.108		0.259	
*λ*_1_′ (s^−1^)	0.037		0.010		0.039		0.066	
*λ*_1_ (s^−1^)	*		*		*		*	
*λ*_2_′ (s^−1^)	3.995		4.708		4.624		3.303	
*λ*_2_ (s^−1^)	3.895		4.608		4.524		3.203	
*λ*_3_′ (s^−1^)	12.525		13.919		12.947		6.675	
*λ*_3_ (s^−1^)	12.425		13.819		12.847		6.575	
*k*_12_ (s^−1^)	6.289		4.663		5.433		6.382	
*k*_21_ (s^−1^)	2.896		4.187		1.928		2.356	
*k*_23_ (s^−1^)	0.406		0.414		0.434		0.359	
*k*_32_ (s^−1^)	0.029		0.028		0.043		0.024	
*ε* (µm)	0.036		0.037		0.033		0.036	
**Data**								
(molecules)	1967		1583		865		504	
(cells)	6		4		10		21	

* not determined. The apparent rate constant, *λ*_1_′, is smaller than that of photobleaching (*λ*_b_ = 0.1 s^−1^), suggesting dissociation from the membrane rarely occurs when the molecule adopts state 1.

# 95% confidence interval for the estimates of diffusion coefficients.

*p_j_* represents the ratio of the *j*-th subpopulation in the displacement data calculated at an arbitrary time in the trajectories.

*q_j_* represents the initial probability that the molecule adopts the *j*-th state upon the onset of membrane association.

The standard deviation of the measurement error, *ε*, was estimated from the trajectories by fitting *MSD*(Δ*t*) from Δ*t* to 2Δ*t*.

In the third step, state transitions are determined by examining alternations in the diffusion mobility of single-molecule trajectories. In order to detect stochastic alternations, the displacement is statistically analyzed according to time after membrane association. We studied trajectories in the time interval between *t* and *t*+τ with τ> = Δ*t*, and calculated the displacement, Δ*r*, within the interval. The displacement distribution for a given interval is a mixture of distributions with diffusion coefficients that are determined by MLE as described above. We then estimated the ratio, *p_j_*, in Eq. 11 for every interval by using the diffusion coefficients. The ratio at the time interval between *t* and *t*+τ is represented by *p_j_*(*t*), which demonstrates the ratio is time dependent (Figure 3C, inset). The decay profiles of the subpopulations are obtained by multiplying the dissociation curve with *p_j_*(*t*) and are expected to follow the subpopulation probabilities *Q*_j_(*t*) obtained theoretically (Figure 3C). In the absence of a state transition, each subpopulation decreases monotonically according to Eq. 6 (Figure 2E). On the other hand, in the presence of a state transition, the probability of each subpopulation shows biphasic decay and finally decreases at the same rate after reaching a steady state with respect to the state transition described by Eq. 9 (Figure 2G) and shown in the analysis of the simulated trajectories (Figure 3C).

Finally, kinetic parameters such as dissociation rate constants, transition rate constants and initial probabilities are quantified by fitting the decay profiles to *Q*_j_(*t*). The parameter values were estimated from the trajectories as *λ*_1_ = 0.11, *λ*_2_ = 0.94, *k*_12_ = 0.068, *k*_21_ = 0.47 and *q*_1_ = 0.20 (Figure 3C), which are in good agreement with the values used in the simulation: *λ*_1_ = 0.10, *λ*_2_ = 1.00, *k*_12_ = 0.10, *k*_21_ = 0.50 and *q*_1_ = 0.20. The PDF of the molecular positions was obtained and compared with the distribution obtained from the molecular trajectories (Figure 3D). When the model PDF fits the experimental distribution well, the estimation is deemed to be successful. According to the same procedure as above, other models could also make successful estimates of the diffusion coefficients and kinetic parameters, including simple models like Models S1 and S2 (Figure S2) as well as more complicated ones like that for PTEN_G129E_ (described below). The estimation accuracy is dependent on the number of trajectories to be analyzed and the magnitude of the measurement error (see Text S1 and Figures S3 and S4).

### State transition and membrane dissociation kinetics of PTEN_G129E_

**Figure 4:**
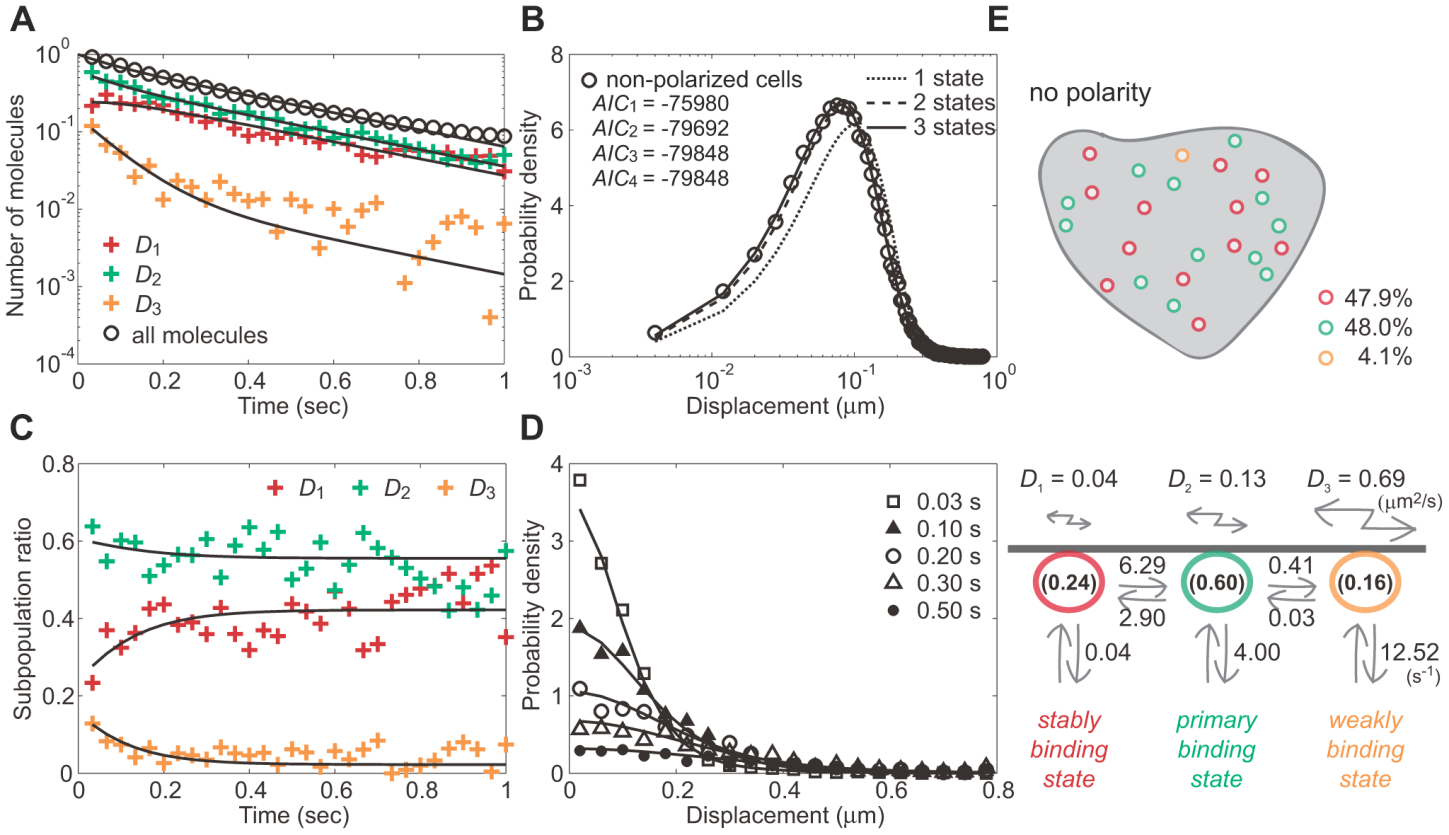
Lifetime-diffusion analysis of PTEN_G129E_ in non-polarized cells. (A) The dissociation curve of all molecules (*open circles*) and decay profiles of three subpopulations (*crosses*) fitted to Eqs. S11 and S12, respectively (*solid lines*). (B) The distribution of displacement during a 33 ms window obtained from the trajectories (*open circles*) and fitting function Eq. 11, assuming 1 (*small dotted line*), 2 (*large dotted line*) or 3 states (*solid line*). (*inset*) AIC values show at least three states are required to explain the data. (C) The time series of the subpopulation ratios. Fitting curves were obtained from Eqs. S11 and S12. (D) The distribution of molecular position at indicated times after the membrane association. Fitting curves were obtained from Eq. S10. (E) Kinetic model describing the state transitions and membrane dissociations in non-polarized cells. All kinetic parameter values estimated in (A)–(D) are summarized in the scheme. See also Movie S2.

Using lifetime-diffusion analysis, we analyzed the trajectories of single PTEN_G129E_ molecules. To elucidate the general properties of membrane interactions that occur independently of cell polarity, molecular behaviors were analyzed in non-polarized cells. The membrane binding duration of each PTEN_G129E_ molecule was measured and the dissociation curve was obtained (Figure 4A, open circles). The decay was faster than the fluorescent photobleaching, indicating transient association of PTEN_G129E_ to the membrane and is consistent with a previous report that examined mammalian and *Dictyostelium* cells [31]. To estimate the state number, the molecular displacement within a 33 ms window was measured to obtain the displacement distribution (Figure 4B). In AIC analysis, the minimum value was obtained by assuming that PTEN_G129E_ adopts three states with different diffusion coefficients (see Text S1 for details). This enabled us to fit well the displacement distribution using Eq. 11 (Figure 4B). Thus, PTEN_G129E_ on the membrane of non-polarized cells likely adopts three states with different diffusion coefficients, *D*_1_ = 0.04, *D*_2_ = 0.13 and *D*_3_ = 0.69 µm^2^s^−1^.

Whether a state transition occurs was determined by quantifying the decay profiles. In Figure 4C, the ratio of each subpopulation, which was obtained using the same manner as that for the inset in Figure 3C, was plotted as a function of time after the onset of membrane association. At the initial moment of membrane association, state 2 dominated the three states. All three ratios changed initially but eventually reached steady state after 500 ms when the ratios became constant. By multiplying the dissociation curve by the ratios, the decay profiles were obtained, showing the same decay rate after 500 ms of membrane association (Figure 4A, crosses). Thus, all three states are likely to be involved in state transitions.

The minimum model assumes three states with different diffusion coefficients and at least two state transitions from a given state (Figure 4E). The diffusion equations for the model are,
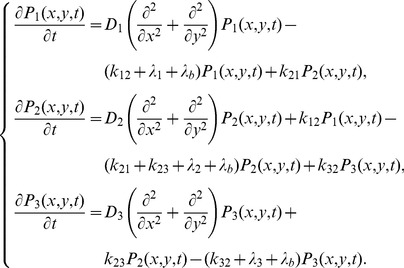
(12) The initial probabilities that the molecule adopts states 1, 2 or 3 are represented by *q*_1_, *q*_2_ and *q*_3_, respectively, with *q*_1_+*q*_2_+*q*_3_ = 1. These equations were solved after taking the Fourier transform, and the inverse transformation was performed to obtain the PDF by numerical integration (Figure 4D). The derivations of *R*(*t*) and *Q*_j_(*t*) are described in Text S1. In Figure 4A, the three decay profiles were well fitted by the theoretical functions with a single set of parameter values, indicating that this model can explain the membrane dissociation and state transition kinetics. The rate constants estimated by the fitting are summarized in Figure 4E and Table 1. By using these values and diffusion coefficients in the theoretical PDF, we could explain the distributions of position (Figure 4D).

The estimated kinetic model can explain the shuttling of PTEN_G129E_ as follows (Figure 4E). The major state that the molecule adopts initially on the membrane is state 2, indicating that state 2 is the primary binding state responsible for recruitment of PTEN_G129E_ to the membrane. When the molecule adopts state 1 by transition from state 2, the rate constant of membrane dissociation decreases by a factor of 100, indicating that state 1 is a stably binding state that extends the membrane association duration. When the molecule adopts state 3 instead, the membrane dissociation rate increases by a factor of 3 compared with state 2. A state 3 to state 2 transition is sufficiently rare such that almost all molecules are returned to the cytoplasm upon adopting state 3, indicating this state is a weakly binding state that accelerates membrane dissociation. Therefore, most PTEN_G129E_ molecules were suggested to be recruited to the membrane primarily via state 2, fluctuate between states 1 and 2, but return to the cytoplasm at the highest probability when they take state 3. The membrane dissociation kinetics are sensitive to changes in *λ*_2_, *k*_12_, *k*_21_ and *q*_1_/*q*_3_ such that the membrane binding lifetime can be modulated through these parameters (Text S1, Figure S5).

**Figure 5:**
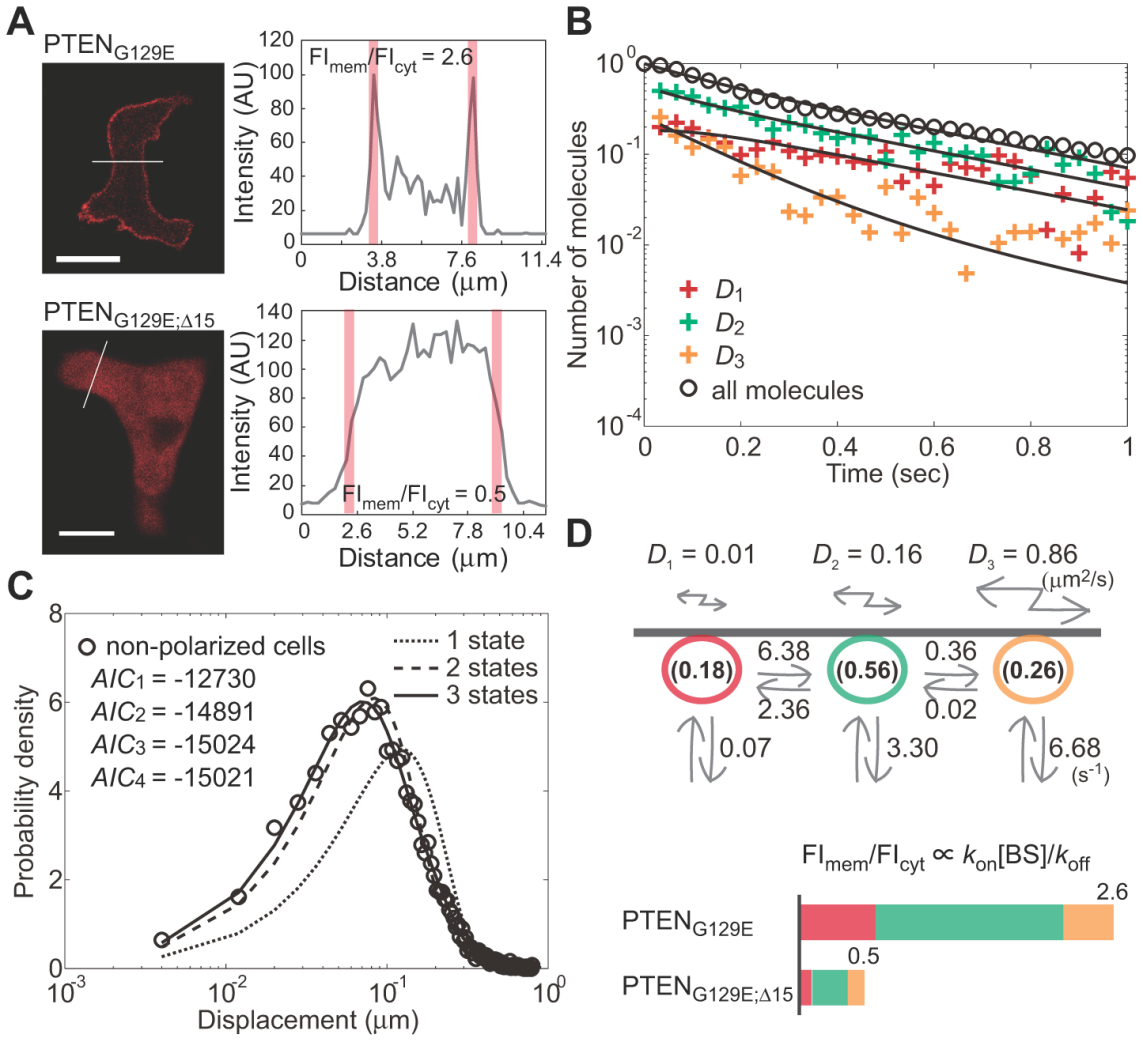
Lifetime-diffusion analysis of PTEN_G129E;Δ15_ in non-polarized cells. (A) Fluorescent images of *Dictyostelium discoideum* cells expressing PTEN_G129E_–Halo (upper panel) or PTEN_G129E;Δ15_–Halo (lower panel) labeled with a TMR-conjugated HaloTag ligand (left). Fluorescence intensity along a white line was measured (right). Red bars indicate the area corresponding to the membranes. Scale bar, 10 µm. (B) The dissociation curve of all PTEN_G129E;Δ15_ molecules (*open circles*) and decay profiles of three subpopulations (*crosses*) fitted to Eqs. S11 and S12, respectively (*solid lines*). (C) The distribution of displacement during a 33 ms window obtained from the trajectories (*open circle*) and fitting function Eq. 11, assuming 1 (*small dotted line*), 2 (*large dotted line*) or 3 states (*solid line*). (*inset*) AIC values show at least three states are required to explain the data. (D) Kinetic model describing the state transitions and membrane dissociation of PTEN_G129E;Δ15_ in non-polarized cells. The difference in membrane-to-cytoplasm ratio of fluorescence intensity is related to the difference in membrane association rate (s^−1^) between PTEN_G129E_ and PTEN_G129E;Δ15_ which dissociated from the membrane at the same rate on average.

It has been suggested that PTEN membrane localization requires the PtdIns(4,5)P_2_-binding motif located at its N-terminus [28], [31], [32]. We therefore considered whether the membrane binding states are dependent on this motif. PTEN_G129E;Δ15_-Halo, in which 15 amino acids constituting the motif were deleted, exhibited more localization to the cytoplasm (Figure 5A). The fluorescence intensity of PTEN_G129E_-Halo showed a 160% increase on the membrane compared to the cytoplasm. On the other hand, PTEN_G129E;Δ15_-Halo on the membrane was 50% that in the cytoplasm (Figure 5A). Consistent with this, the number of PTEN_G129E;Δ15_ molecules observed on the membrane was largely decreased. The dissociation curve was not altered by deletion of the motif, indicating that the cytoplasmic localization of PTEN_G129E;Δ15_-Halo was mainly due to a decrease in the membrane association rate rather than an increase in the membrane dissociation rate (Figures 4A and 5B). Lifetime-diffusion analysis suggested the most likely state number for PTEN_G129E;Δ15_-Halo is 3, and diffusion coefficients and kinetic parameters similar to those of PTEN_G129E_ were estimated (Figures 5C and 5D, Table 1). Furthermore, the initial probabilities also resembled those of PTEN_G129E_. These results suggest that the PtdIns(4,5)P_2_-binding motif is a prerequisite for membrane association independent of the state.

### Heterogeneity in state transition kinetics of polarized cells

Based on the model for non-polarized cells, stochastic trajectories in polarized cells were analyzed to reveal the mechanism for how heterogeneity in molecular behavior is established. AIC analysis was performed using the displacement distributions obtained from the pseudopod and tail membrane, with each location showing three states (Figure 1F). The diffusion coefficients were estimated to be 0.04, 0.11 and 0.56 µm^2^s^−1^ at the pseudopod, and 0.03, 0.15 and 0.72 µm^2^s^−1^ at the tail. These values were similar to those estimated in non-polarized cells, indicating that the diffusion coefficient of each state was not affected significantly by cell polarity. We did observe that the two regions showed differences in the relative amount of each subpopulation on the membrane: PTEN_G129E_ adopts state 1, the stably binding state, less frequently at the pseudopod (34%) than at the tail (64%), which leads to an increase in average diffusion mobility (Figure 1F).

**Figure 6:**
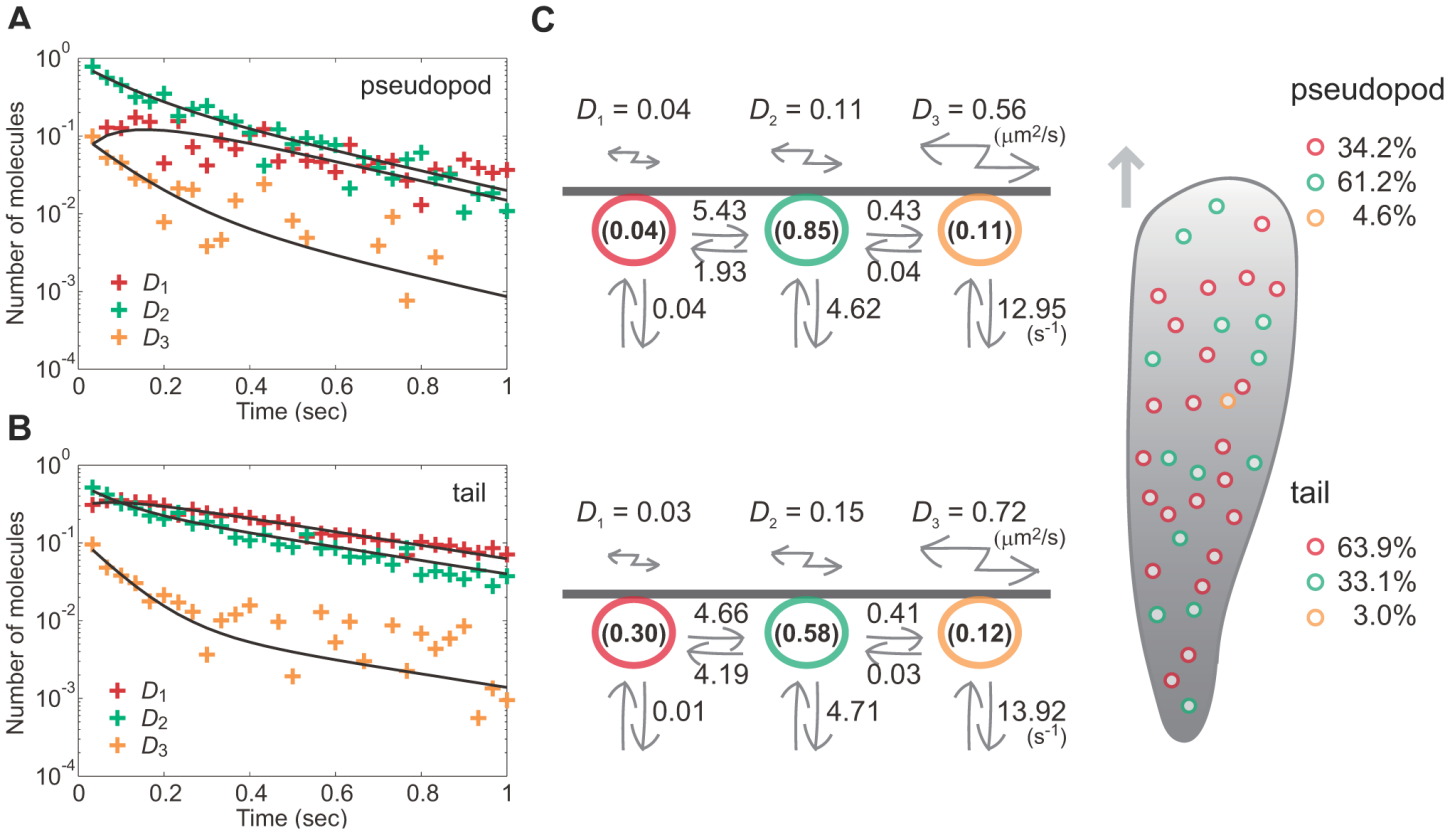
Lifetime-diffusion analysis of PTEN_G129E_ in polarized cells. (A) The decay profiles of three subpopulations obtained from molecules observed at the pseudopod (*crosses*) and fitted to Eq. S12 (*solid lines*). (B) The decay profiles of three subpopulations obtained from molecules observed at the tail (*crosses*) and fitted to Eq. S12 (*solid lines*). (C) Kinetic model describing the state transitions and membrane dissociation in polarized cells. All kinetic parameter values estimated in (A) and (B) are summarized in the scheme. See also Movie S1.

The decay profiles showed that PTEN_G129E_ on both regions exhibits state transitions (Figures 6A and 6B). All subpopulations decayed at the same rate after reaching the stationary state. At the onset of membrane association, the major state was state 2 irrespective of cell polarity. After 500 ms of membrane association, state 2 remained the major state in the pseudopod, whereas it was state 1 in the tail (Figures 6A and 6B). These results indicate that the state transition kinetics of PTEN_G129E_ molecules is different between the pseudopod and tail of polarized cells.

We estimated the rate constants and initial probabilities by fitting the decay profiles (Figure 6C, Table 1). The dissociation rate constants for both regions were almost equivalent to the respective rate constants of non-polarized cells, indicating that cell polarity does not significantly affect the membrane-binding ability of each state. However, a remarkable change was found in the ratios of the states that the molecule adopts on its initial moment of membrane association (Figure 6C). Initially at the pseudopod, state 1 PTEN_G129E_ molecules were few (4%), while at the tail they exceeded those seen in non-polarized cells (30% vs. 24%). Consistent with this, the transition from state 2 to 1 was decelerated at the pseudopod (*k*_21_ = 1.93), but accelerated at the tail (*k*_21_ = 4.19) relative to non-polarized cells (*k*_21_ = 2.90). On the other hand, the transition from state 1 to 2 occurred slightly faster at the pseudopod (*k*_12_ = 5.43) than at the tail (*k*_12_ = 4.66). As a result, the equilibrium between states 1 and 2 is biased toward state 2 at the pseudopod and toward state 1 at the tail compared to non-polarized cells. These results indicate that the membrane binding of PTEN_G129E_ is suppressed at the pseudopod, but stabilized at the tail, a property that can explain, on average, the spatial heterogeneities in membrane-binding lifetime and lateral mobility. Because the transition rates between state 2 and 3 are constant and independent of cell polarity, state 1, the stably binding state, should play a major role in regulating the membrane-binding properties of a polarized cell.

### Asymmetric distribution based on model kinetics

**Figure 7:**
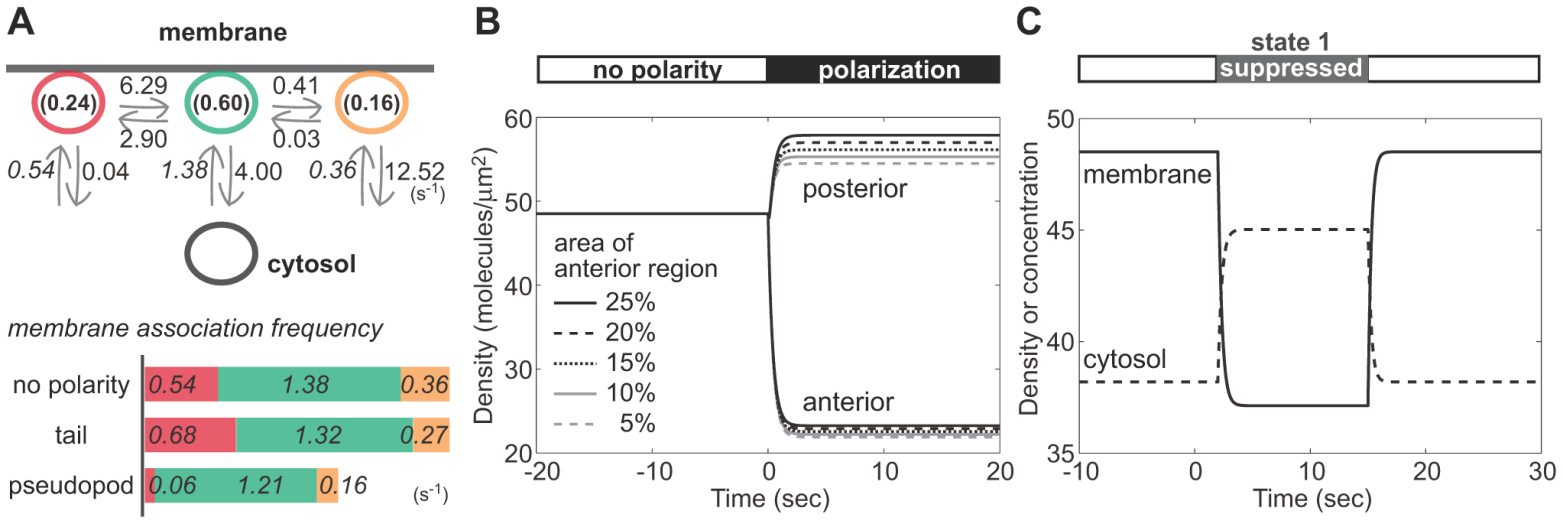
Simulations of the intracellular distribution. (A) Estimation of membrane association frequencies that a single cytoplasmic molecule associates with the membrane during 1 sec via three states. (B) The density of membrane bound molecules in the absence or presence of cellular polarity. (C) A temporal change in the density on the membrane (molecules/µm^2^) and the concentration in the cytosol (molecules/µm^3^) as *μ*_1_ approaches 0 during *t* = 2 to 15 sec.

In order to examine whether differences in the kinetic parameters sufficiently explain the depletion of PTEN from the anterior pseudopod, we performed numerical simulations of the intracellular distribution based on the above model using experimentally obtained kinetic parameters. For this purpose, the membrane association frequency, which represents the frequency that a single molecule in the cytoplasm associates with the membrane in 1 sec, was estimated (Figure 7A). Here we assumed that the membrane localization is in steady state in non-polarized cells of a constant volume and reaches a constant membrane PTEN to cytoplasm PTEN ratio in the molecular number. The ratio was quantified biochemically from the ensemble fluorescence intensities of TMR-labeled PTEN_G129E_-Halo in insoluble and soluble fractions of the cell lysate [33]. The ratio was 0.76 and gave rise to three membrane association frequencies: *μ*_1_ = 0.54, *μ*_2_ = 1.38 and *μ*_3_ = 0.36 s^−1^, since the association frequencies are proportional to the respective initial probabilities, *q*_1_, *q*_2_ and *q*_3_ (Figure 7A, Text S1). In the simulation where the number of cytosolic molecules was 20000 and the radius of the cell was spherical and 5 µm, the total number of membrane-bound molecules was 15200 and the density on the membrane was 48.5 molecules/µm^2^ (time<0 s in Figure 7B).

Next, the association frequencies at the pseudopod and tail were estimated (see Text S1 for details). By directly counting the number of molecules that began their membrane association within a unit time interval in a unit area at the pseudopod or tail of a single cell, we found the overall association frequency via all three states, *μ* = *μ*_1_+*μ*_2_+*μ*_3_, at the pseudopod to be on average approximately 60% that of the tail (n = 5 cells). When *μ* at the tail was assumed to equal the *μ* of non-polarized cells, the association frequencies were *μ*_1_ = 0.68, *μ*_2_ = 1.32 and *μ*_3_ = 0.27 s^−1^ at the tail and *μ*_1_ = 0.06, *μ*_2_ = 1.21 and *μ*_3_ = 0.16 s^−1^ at the pseudopod (Figure 7A, see Text S1 for detail). At time 0 in Figure 7B, when the kinetic parameters obtained in the polarized cells were incorporated, the simulated PTEN density on the anterior and posterior membrane diverged to show remarkable depletion from the anterior membrane, which recapitulates the asymmetry that arose with a cAMP gradient (Figure 1B). The calculated posterior-to-anterior ratio of the PTEN density reached 2.5, which is comparable to the ratio measured experimentally by the fluorescent imaging of PTEN-Halo (2.1). Thus, PTEN is likely to be excluded from the pseudopod membrane of chemotaxing cells, probably due to the suppression of molecules adopting state 1, the stable binding state. Consistently, when membrane recruitment via state 1 was temporally inhibited in a step-wise manner in a non-polarized cell, the simulated PTEN density on the membrane decreased quickly within 1.5 s after the change (Figure 7C), demonstrating the importance of state 1 in regulating the amount of membrane-bound PTEN molecules. The suppression of state 1 then is likely to be a key regulator for the asymmetric PTEN distribution.

### Parallel analysis between two-state and three-state models

**Figure 8:**
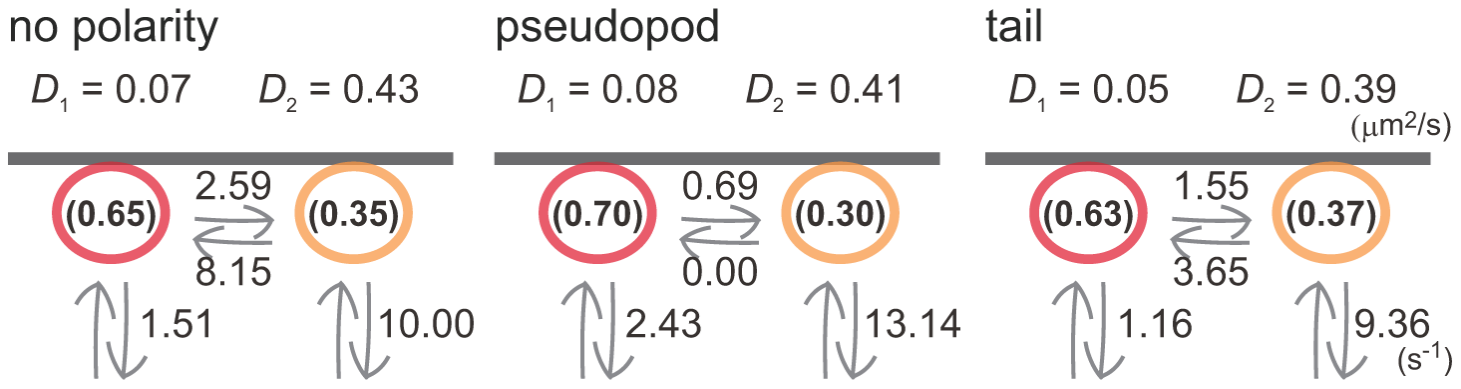
Lifetime-diffusion analysis assuming a two-state model. Kinetic model describing the state transitions and membrane dissociations in non-polarized cells and at the pseudopod and tail of polarized cells. All kinetic parameter values estimated are summarized in the scheme.

We also performed lifetime-diffusion analysis assuming PTEN_G129E_ molecules adopt two states, because the PDF of displacement with two diffusion coefficients was similar to that with three diffusion coefficients when fitting the experimental data (Figure 4B). The kinetics of the state transitions and membrane dissociation were analyzed from the experimental trajectories. In non-polarized cells, the estimated *D*s were 0.07 and 0.43 µm^2^/sec for states 1 and 2, respectively. The probabilities of adopting these states decayed at the same rate after 300 to 500 msec of membrane association, suggesting the molecules exhibit state transitions (Figure S6). By fitting the decay profiles to Eq. 9, the kinetic parameters were estimated as *k*_12_ = 2.59, *k*_21_ = 8.15, *λ*_1_ = 1.51, *λ*_2_ = 10.00 and *q*_1_ = 0.65 (Figure S6). The same analyses were performed on the trajectories obtained for polarized cells (Table 2, Figure 8). The two models did share some essential kinetic features. The state with slower lateral diffusion exhibited slower membrane dissociation, and transition from the faster state to slower state was inhibited at the pseudopod. As a result, PTEN molecules that once adopted the faster state readily dissociated from the membranes at the pseudopod, resulting in shorter membrane binding lifetimes and slower lateral diffusion there (Figures 1E and 1F). However, while a low initial probability of state 1 at the pseudopod was suggested in the three-state model, the initial probability in the two-state model was relatively unaffected by cell polarity. It is possible that states 1 and 2 in the two-state model behave as averages of states 1 and 2 and states 2 and 3, respectively, in the three-state model. Therefore, the two-state model may serve as an average model, while the three-state model, which seems better able to statistically explain the trajectories obtained experimentally (Text S1, Figure S7), is more accurate.

**Table 2:** Estimated parameters (two-state model).

	PTEN_G129E_						PTEN_G129E;Δ15_	
	No polarity		Posterior		Anterior		No polarity	
**Parameters**		CI#		CI#		CI#		CI#
*D*_1_ (µm^2^s^−1^)	0.067	0.064	0.052	0.050	0.077	0.072	0.057	0.051
		0.069		0.055		0.082		0.063
*D*_2_ (µm^2^s^−1^)	0.435	0.402	0.389	0.359	0.406	0.334	0.551	0.504
		0.468		0.419		0.477		0.600
*p*_1_	0.884		0.878		0.902		0.737	
*p*_2_	0.116		0.122		0.098		0.263	
*q*_1_	0.646		0.625		0.697		0.512	
*q*_2_	0.354		0.375		0.303		0.488	
*λ*_1_′ (s^−1^)	1.510		1.160		2.431		1.131	
*λ*_1_ (s^−1^)	1.410		1.060		2.331		1.031	
*λ*_2_′ (s^−1^)	9.998		9.359		13.142		6.252	
*λ*_2_ (s^−1^)	9.898		9.259		13.042		6.152	
*k*_12_ (s^−1^)	2.592		1.553		0.688		0.966	
*k*_21_ (s^−1^)	8.155		3.653		0.000		0.000	

# 95% confidence interval for the estimates of diffusion coefficients.

## Discussion

Here we describe a statistical model that uses single-molecule imaging analysis to explain the molecular mechanism responsible for the asymmetric membrane localization of PTEN. By developing and using a novel method, lifetime-diffusion analysis, we propose a multistate kinetics model in which PTEN_G129E_ on the membrane has three membrane-binding states that transit between themselves. The model can explain the heterogeneity of PTEN molecular behavior as the result of suppressing the stable binding state at the pseudopod. A simulation based on the model demonstrated that locally applied modulation on the stable binding state gives rise to an asymmetric distribution of PTEN on the membrane, suggesting an essential causal relationship between molecular membrane-binding kinetics and the PTEN distribution on the membrane of polarized cells.

We compare the results obtained by the lifetime-diffusion analysis with those from lifetime or diffusion analysis. The dissociation curve of PTEN_G129E_ in non-polarized cells was fitted to a sum of three exponential functions, Eq. S11, with rate constants of 2.5 (95% in the relative amount), 11.1 (3%) and 12.6 s^−1^ (2%). The distribution of the displacement obtained from the same trajectories was fitted to a sum of three PDFs, Eq. 11, with diffusion coefficients of 0.04 (48%), 0.13 (48%) and 0.69 (4%) µm^2^s^−1^. Thus, analysis of membrane-binding and diffusion mobility led to different proportions of the subpopulations of the three states. In other words, temporal and spatial analysis gives different results for the three states. In contrast, our lifetime-diffusion analysis method offers a quantitative estimate of the kinetic parameter for each state simultaneously with the motility parameter. Applying the method to the trajectories of PTEN_G129E_ successfully provides a multistate kinetics model by which the spatiotemporal properties of the single-molecule trajectories can be quantitatively explained.

Three membrane-binding states were found to have different roles for regulating the shuttling of PTEN_G129E_ molecules between the membrane and cytoplasm. The majority of molecules adopt state 2 at the onset of membrane association. Molecules that adopt this state can proceed in one of three ways: dissociation from the membrane (highest probability), transition into state 1 or transition into state 3 (lowest probability). By transforming into state 1, the membrane association lifetime increased by about 100-fold. Transition into state 3 had the reverse effect, speeding dissociation. Thus, the intracellular distribution of PTEN_G129E_ is regulated via the state transition that occurs on the membrane, and the asymmetric distribution of PTEN located there is dependent on local differences in the state transition kinetics. The lifetime of intact PTEN was also shorter at the pseudopod than at the tail, suggesting that the same mechanism should principally work in localizing intact PTEN molecules [34].

The diffusion coefficients of molecules on the membrane depend mainly on their interactions, with the coefficients generally becoming smaller as the interaction interface becomes larger. For example, proteins associating only with the inner leaflet usually have a larger diffusion coefficient than those integrated into the lipid bilayer [35]. In the case of *Dictyostelium* cells, CRAC, a pleckstrin homology (PH) domain-containing protein which can bind to PtdIns(3,4,5)P_3_ on the inner leaflet, has a diffusion coefficient of 0.14 µm^2^s^−1^ [9], while the diffusion coefficient of Gβγ molecules that bind to the membrane via isoprenylation is 0.096 µm^2^s^−1^ [36]. On the other hand, the cAMP receptor cAR1, a seven transmembrane receptor, has a diffusion coefficient of 0.022 µm^2^s^−1^ [8], [34]. Referring to these known values, we propose that states 2 and 3 only occur on the inner leaflet. Also of note is that state 2 shows a diffusion coefficient (0.11 to 0.15 µm^2^s^−1^) which is similar to that of the PtdIns(3,4,5)P_3_-binding protein CRAC. Since the membrane association of PTEN requires a N-terminal PtdIns(4,5)P_2_-binding motif (Figure 5) [28], [31], [32], state 2 likely involves binding to PtdIns(4,5)P_2_. On the other hand, state 1 presumably depends on some membrane-integral protein in addition to PtdIns(4,5)P_2_, although such a binding partner for PTEN has not been identified in *Dictyostelium* to date. This putative binding partner seems to be a key regulator for PTEN dynamics and thus for asymmetry generation in chemotactic signaling.

We have previously reported that PTEN and PtdIns(3,4,5)P_3_ exhibit mutually exclusive membrane localizations in the absence of a cAMP gradient and in the presence of Latrunculin A and caffeine, and proposed a reaction-diffusion model assuming PtdIns(3,4,5)P_3_ negatively regulates PTEN membrane localization [22], [37]. To account for such a regulation, the high PtdIns(3,4,5)P_3_ concentration on the membrane should change the membrane interaction kinetics of PTEN, leading to one or both of a decrease in the membrane association rate and an increase in the membrane dissociation rate. We found that the association was less frequent and the dissociation was faster at the pseudopod than at the tail by single molecule imaging, and it was suggested by lifetime-diffusion analysis that both are due at least in part to the inhibition of stably binding state. It is plausible that the aforementioned putative binding partner is inhibited by PtdIns(3,4,5)P_3_. Therefore, the three-state kinetics should be examined after modulating PtdIns(3,4,5)P_3_ levels on the membrane. In addition, the negative regulation requires phosphatase activity of PTEN in the spontaneous formation of the PtdIns(3,4,5)P_3_ domain, which could be further examined by observing intact PTEN molecules.

In conclusion, lifetime-diffusion analysis provides a multistate kinetic description of intracellular signaling without *a priori* information of the interaction partner molecules, and can therefore be readily applied to any type of single-molecule trajectory.

## Materials and Methods

### Cell strain

*Dictyostelium discoideum* wild-type strain Ax2 was used as the parental strain. The plasmid was generated from the extrachromosomal expression of HaloTag-fusion PTEN proteins (PTEN-Halo) (Promega, Japan) and introduced into Ax2 cells by electroporation [38]. A point mutation substituting E for G at amino acid 129 (G129E) was introduced according to the protocol of the QuikChange site-directed mutagenesis kit (Agilent Technologies).

### TIRFM observation

The observation of single PTEN molecules was performed using cells prepared as follows. Cultured cells were suspended at 3×10^6^ cells/ml in development buffer (DB; 5 mM Na_2_HPO_4_, 5 mM NaH_2_PO_4_, 2 mM MgSO_4_, 0.2 mM CaCl_2_, pH 6.2). 1 mL of the cell suspension was transferred to a 35-mm culture dish and kept still for about 7 hours at 21°C. When cells began to aggregate, HaloTag tetramethylrhodamine (TMR) ligand (Promega, Japan) was added to the cell suspension at a final concentration of 50 nM. After 10 minutes of incubation, DB was replaced with new DB twice to rinse out the residual ligands. The cells were washed twice with DB by centrifugation and suspended in DB at around 5×10^6^ cells/ml. 10 µL of the cell suspension was placed on a glass coverslip and the cells were left to settle in a moist chamber. After 10-minute incubation, the cells were overlaid with a 1 cm square sheet of agarose (2% Agarose M in DB) and excess DB was removed [39]. After 20 to 30 min and recommencement of cell aggregation, cells were observed under TIRFM.

Single PTEN-Halo molecules labeled with TMR were observed under an objective type TIRFM constructed on an inverted fluorescence microscope (IX70, Olympus, Japan). The detailed configuration of the microscope system is described elsewhere [16].

### Single-molecule tracking and statistical analysis

The trajectories of single molecules exhibiting lateral diffusion on the membrane were obtained semi-automatically from the onset of movement until their completion using laboratory-made software. Briefly, the position of a single molecule was determined in each frame of a movie by fitting the fluorescence intensity profile to a two-dimensional Gaussian distribution. The positions of neighboring frames were regarded as part of a single trajectory when the distance was within a value typical for diffusing molecules [35].

### Numerical simulations

Trajectories of single molecules were made by numerical simulations previously described [13]. Assuming a Brownian particle, a time series of the position, (*x*(*t*), *y*(*t*)), beginning at the origin was generated from *t* = 0 to 60 s. The simulated time series consisted of 18001 time steps with an interval of 1/300 s. A trajectory of 1801 time steps was obtained by extracting the data from the time series at a time interval of 1/30 s. In the trajectory, a Gaussian SD of 40 nm was added to the position at every time point. Photobleaching of the fluorophore was not taken into account. 3000 trajectories were used for each analysis unless noted otherwise.

## Supporting Information

Figure S1 **Single-molecule imaging of PTEN_G129E_-Halo-TMR molecules in non-polarized cells.** (A) Histograms of fluorescence intensity of PTEN_G129E_-Halo-TMR molecules observed in a single cell (lines, 4 cells). The histogram shown with a thick line is taken from all the molecules observed in 6 cells. (*inset*) Fluorescence emission from a single TMR molecule conjugated to PTEN_G129E_-Halo. (B) Mean square displacement (MSD) of PTEN_G129E_-Halo-TMR molecules. MSD calculated from the trajectories of 15 individual molecules observed for at least 6 sec was plotted against time. (*inset*) Estimation of the measurement error using averaged MSD. The standard deviation of the error was 47 nm (*triangles*, PTEN_G129E_ in non-polarized cells) and 49 nm (*crosses*, PTEN_G129E_ in fixed cells).
(TIF)

Figure S2 **Lifetime-diffusion analysis of the trajectories generated by numerical simulations.** (A,D) Histograms of displacement, Δ*r*, during a time interval of Δ*t* = 0.033. (B,E) Dissociation curves. (C,F) Histograms of position, *x*(*t*), at *t* = 0.4, 0.8, 1.2, 1.6 and 2.0 s (C) or 1, 2, 3, 4 and 5 s (F). (A–C) Model S1. (D–F) Model S2.
(TIF)

Figure S3 **Parameter estimation.** (A–C) Model S1. (D–I) Model S2. (J–Q) Model S3. (A,D,E,J,K) Diffusion coefficient. (B,F,G,L,M) Dissociation rate constant. (C,I,Q) Variance of the measurement error. (H,P) Initial probability of adopting state 1. (N, O) Transition rate constants.
(TIF)

Figure S4 **Estimation of similar parameter values.** Molecular trajectories were generated by numerical simulation assuming Model S3 and analyzed using the lifetime-diffusion analysis method. The estimated parameter values of 10 independent analyses were plotted as relative values to the actual parameter values used in the simulation, which are indicated in the bottom of each panel. 3000 trajectories were used in (C), and 1000 trajectories in the others. (A) Two states with the same dissociation rate constant. (B,C) Two states with similar dissociation rate constants. (D–F) Two states with similar diffusion coefficients (*D*_2_ = 5*D_1_). (G–I) Two different states with similar diffusion coefficients (*D*_2_ = 2*D_1_). Parameter values used in the simulation are indicated in each panel.
(TIF)

Figure S5 **Parameter sensitivity of the three-state model for PTEN_G129E_.** The dissociation curve and decay profiles of the three-state model for PTEN_G129E_ in non-polarized cells are shown after slightly increasing or decreasing each parameter. The changes applied were −10% (blue), −5% (green), 5% (yellow) or 10% (red) (A to H), and −20% (blue), −10% (green), 10% (yellow) or 20% (red) (I). The plots in black are those with original parameter values (Table 1). (A) *λ*_1_. (B) *λ*_2_. (C) *λ*_3_. (D) *k*_12_. (E) *k*_21_. (F) *k*_23_. (G) *k*_32_. (H) *q*_2_. (I) *q*_1_/*q*_3_.
(TIF)

Figure S6 **Lifetime-diffusion analysis of PTEN_G129E_ assuming two states.** The dissociation curve of all molecules (*open circles*) and decay profiles of two subpopulations (*crosses*) fitted to Eqs. 8 and 9, respectively (*solid lines*). (A) Non-polarized cells. (B) Pseudopod. (C) Tail.
(TIF)

Figure S7 **Discrimination between the two- and three-state models.** 2000 trajectories were generated by numerical simulation assuming a three-state (A, B) or two-state model (C, D) for PTEN_G129E_ in non-polarized cells and analyzed. (A, C) AIC values calculated assuming 1 to 4 states with different diffusion coefficients. The results shown in red indicate wrong estimation of the state number. (B, D) The estimated parameter values of 10 independent trials of the simulation and analysis were plotted as relative values to the actual parameter values used in the simulation (Tables 1 and 2). The results shown in red in (B) indicate the estimates from the simulation assuming state 2 (see Text S1).
(TIF)

Movie S1 **Single-molecule imaging of PTEN_G129E_-Halo-TMR in a chemotaxing** ***Dictyostelium discoideum*** **cell.** PTEN_G129E_ molecules were visualized on the basal membrane of the cell moving rightward to the cAMP source.
(MP4)

Movie S2 **Single-molecule imaging of PTEN_G129E_-Halo-TMR in a non-polarized** ***Dictyostelium discoideum*** **cell.** PTEN_G129E_ molecules were visualized in a cell showing uniform membrane localization in the absence of a cAMP gradient.
(MP4)

Text S1 **Supporting information.**
(DOC)
