# Supplementary figures and images for: Asymmetric PTEN Distribution Regulated by Spatial Heterogeneity in Membrane-Binding State Transitions
Source: PLoS Comput Biol. 2013 Jan 10;9(1):e1002862. doi: 10.1371/journal.pcbi.1002862 (PMC3542079; doi:10.1371/journal.pcbi.1002862)

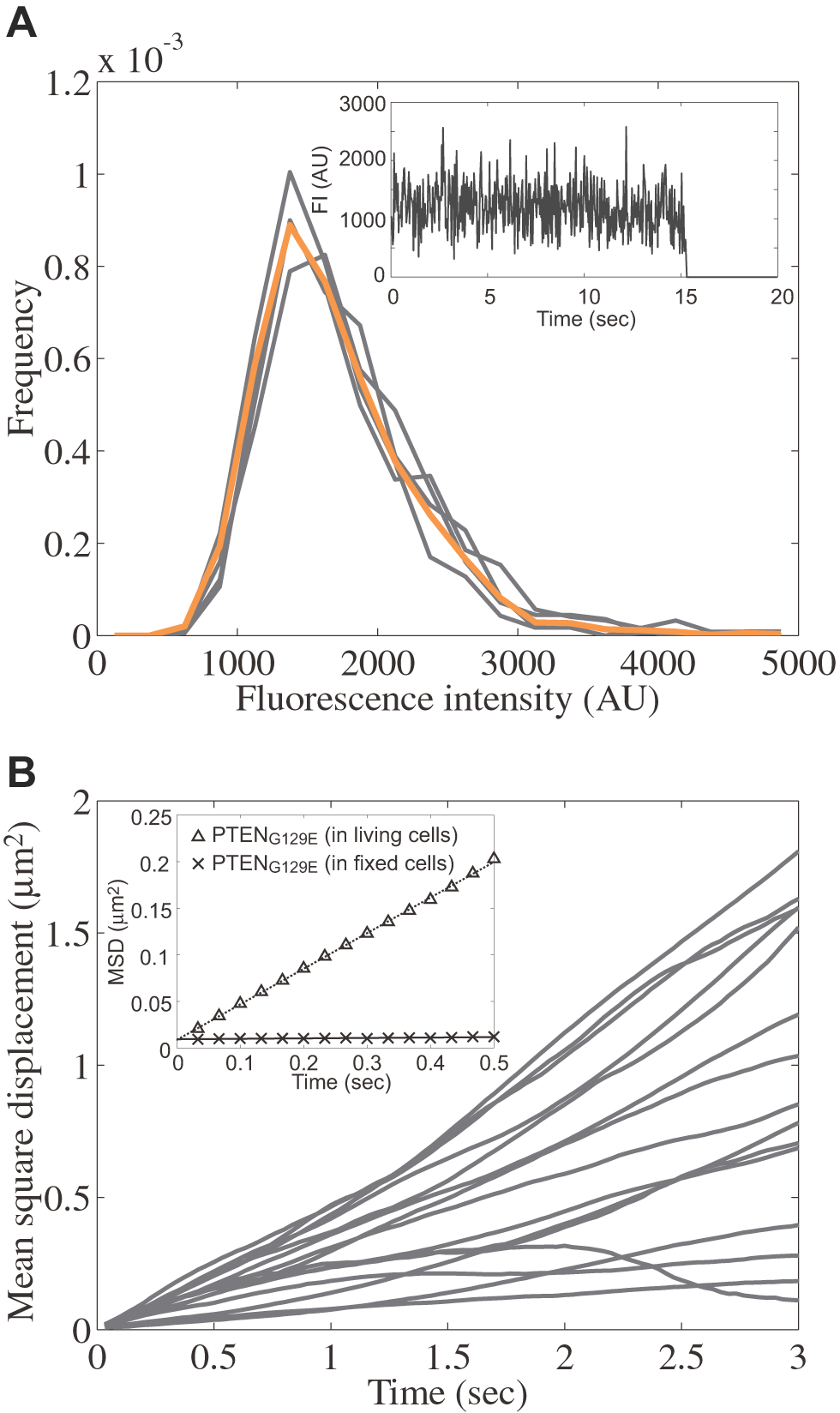

Supplement: Figure S1 — Single-molecule imaging of PTENG129E-Halo-TMR molecules in non-polarized cells. (A) Histograms of fluorescence intensity of PTENG129E-Halo-TMR molecules observed in a single cell (lines, 4 cells). The histogram shown with a thick line is taken from all the molecules observed in 6 cells. (inset) Fluorescence emission from a single TMR molecule conjugated to PTENG129E-Halo. (B) Mean square displacement (MSD) of PTENG129E-Halo-TMR molecules. MSD calculated from the trajectories of 15 individual molecules observed for at least 6 sec was plotted against time. (inset) Estimation of the measurement error using averaged MSD. The standard deviation of the error was 47 nm (triangles, PTENG129E in non-polarized cells) and 49 nm (crosses, PTENG129E in fixed cells). (TIF) [file pcbi.1002862.s001.tif]

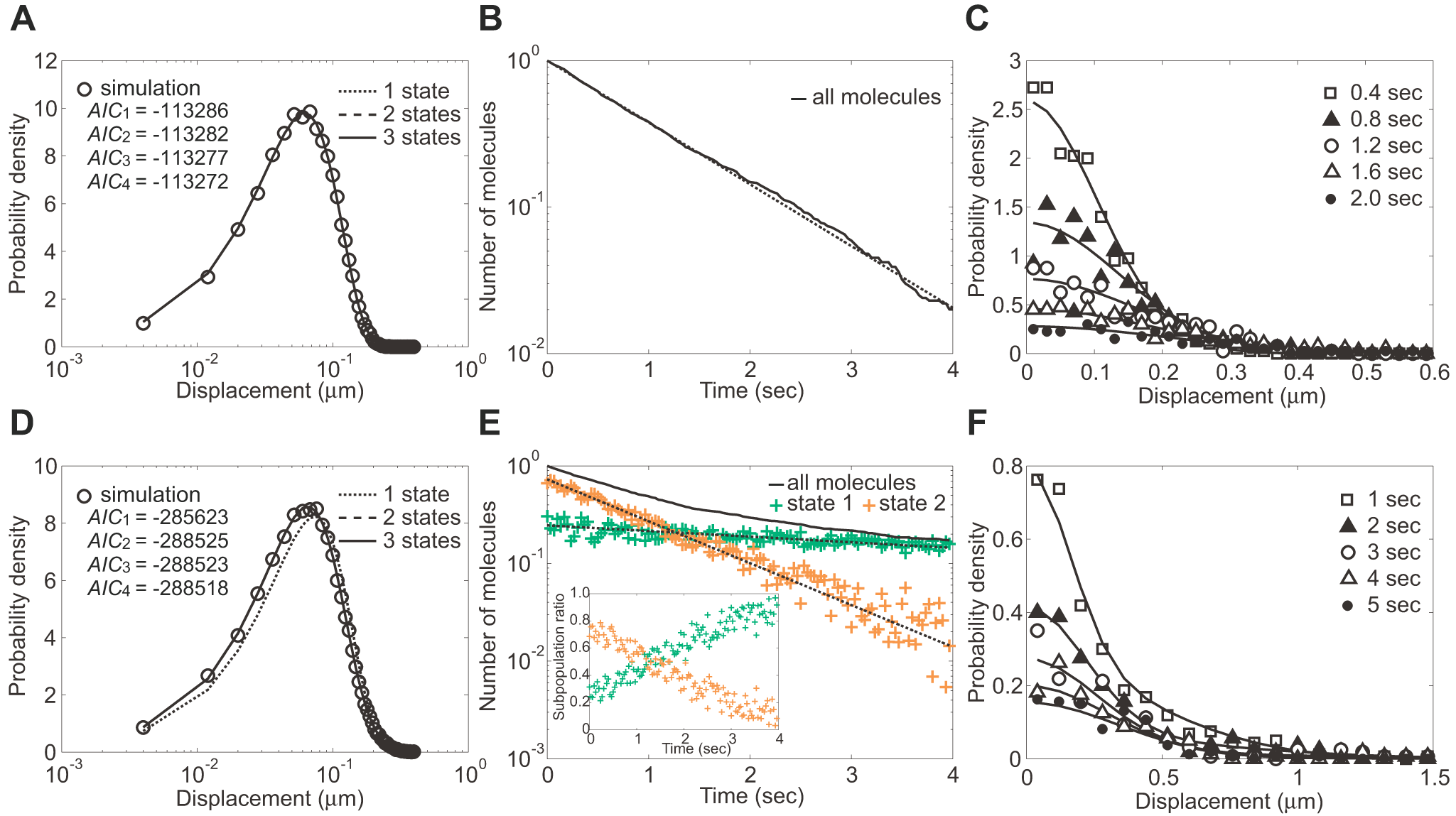

Supplement: Figure S2 — Lifetime-diffusion analysis of the trajectories generated by numerical simulations. (A,D) Histograms of displacement, Δr, during a time interval of Δt = 0.033. (B,E) Dissociation curves. (C,F) Histograms of position, x(t), at t = 0.4, 0.8, 1.2, 1.6 and 2.0 s (C) or 1, 2, 3, 4 and 5 s (F). (A–C) Model S1. (D–F) Model S2. (TIF) [file pcbi.1002862.s002.tif]

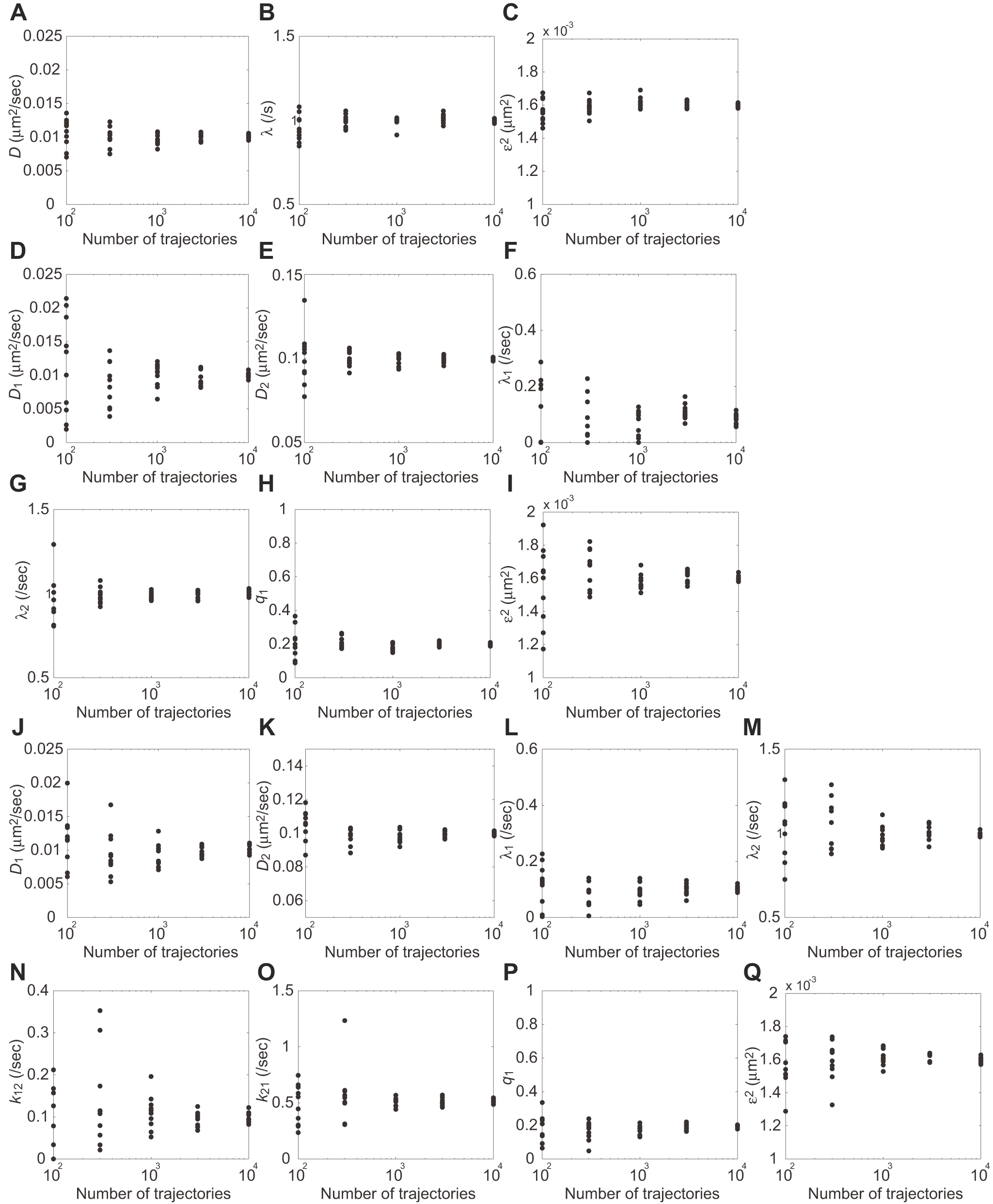

Supplement: Figure S3 — Parameter estimation. (A–C) Model S1. (D–I) Model S2. (J–Q) Model S3. (A,D,E,J,K) Diffusion coefficient. (B,F,G,L,M) Dissociation rate constant. (C,I,Q) Variance of the measurement error. (H,P) Initial probability of adopting state 1. (N, O) Transition rate constants. (TIF) [file pcbi.1002862.s003.tif]

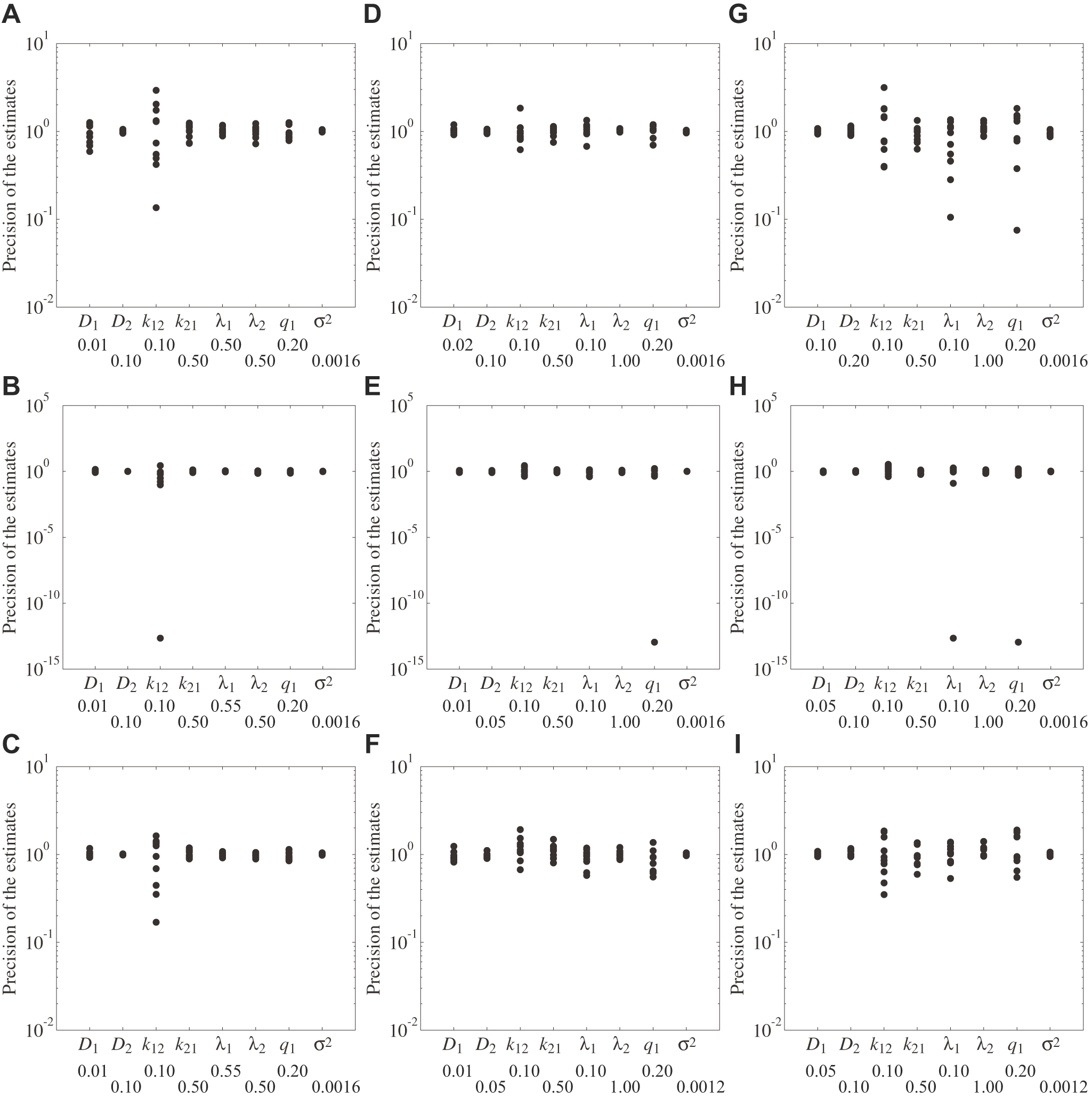

Supplement: Figure S4 — Estimation of similar parameter values. Molecular trajectories were generated by numerical simulation assuming Model S3 and analyzed using the lifetime-diffusion analysis method. The estimated parameter values of 10 independent analyses were plotted as relative values to the actual parameter values used in the simulation, which are indicated in the bottom of each panel. 3000 trajectories were used in (C), and 1000 trajectories in the others. (A) Two states with the same dissociation rate constant. (B,C) Two states with similar dissociation rate constants. (D–F) Two states with similar diffusion coefficients (D 2 = 5*D 1). (G–I) Two different states with similar diffusion coefficients (D 2 = 2*D 1). Parameter values used in the simulation are indicated in each panel. (TIF) [file pcbi.1002862.s004.tif]

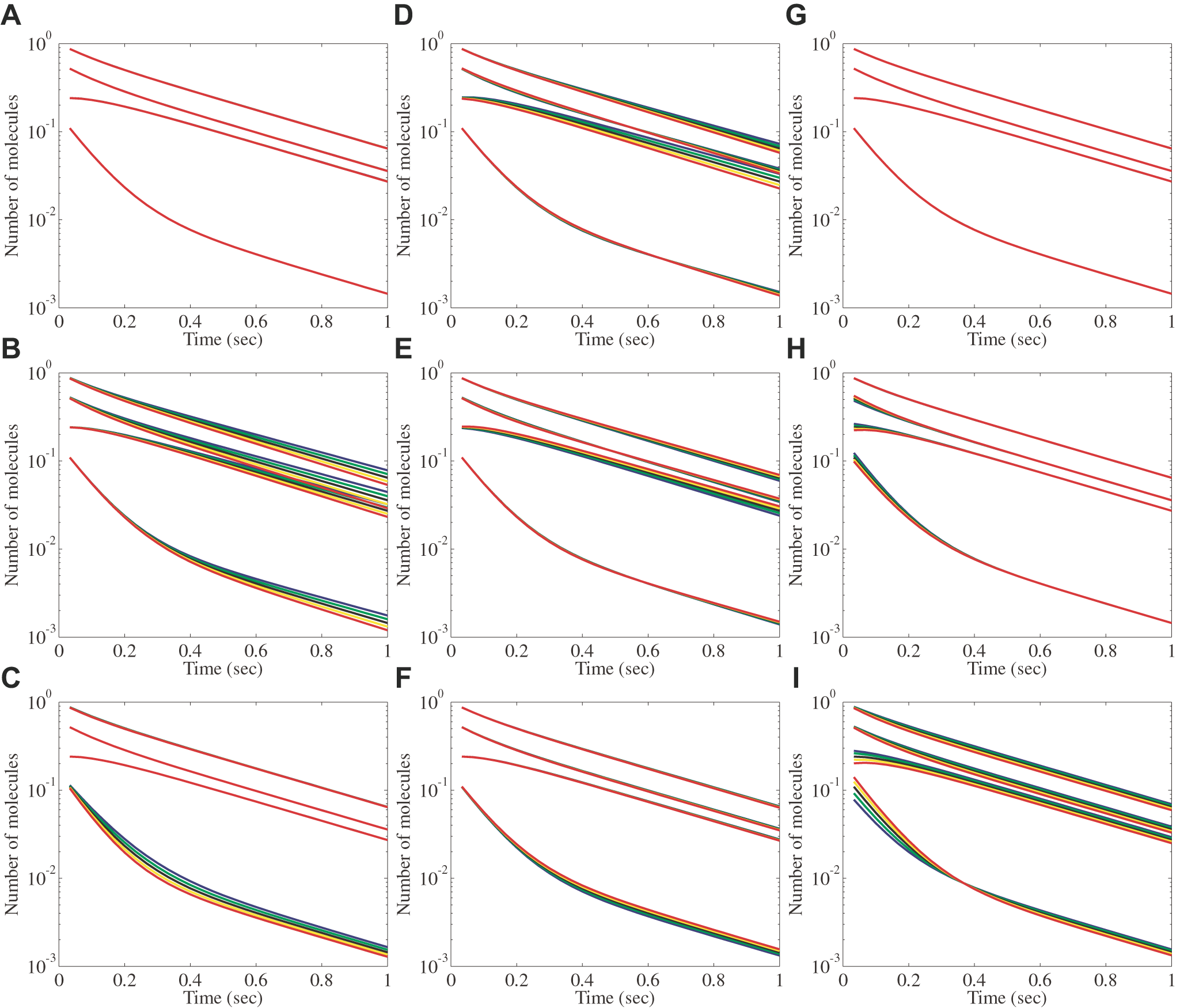

Supplement: Figure S5 — Parameter sensitivity of the three-state model for PTENG129E. The dissociation curve and decay profiles of the three-state model for PTENG129E in non-polarized cells are shown after slightly increasing or decreasing each parameter. The changes applied were −10% (blue), −5% (green), 5% (yellow) or 10% (red) (A to H), and −20% (blue), −10% (green), 10% (yellow) or 20% (red) (I). The plots in black are those with original parameter values (Table 1). (A) λ 1. (B) λ 2. (C) λ 3. (D) k 12. (E) k 21. (F) k 23. (G) k 32. (H) q 2. (I) q 1/q 3. (TIF) [file pcbi.1002862.s005.tif]

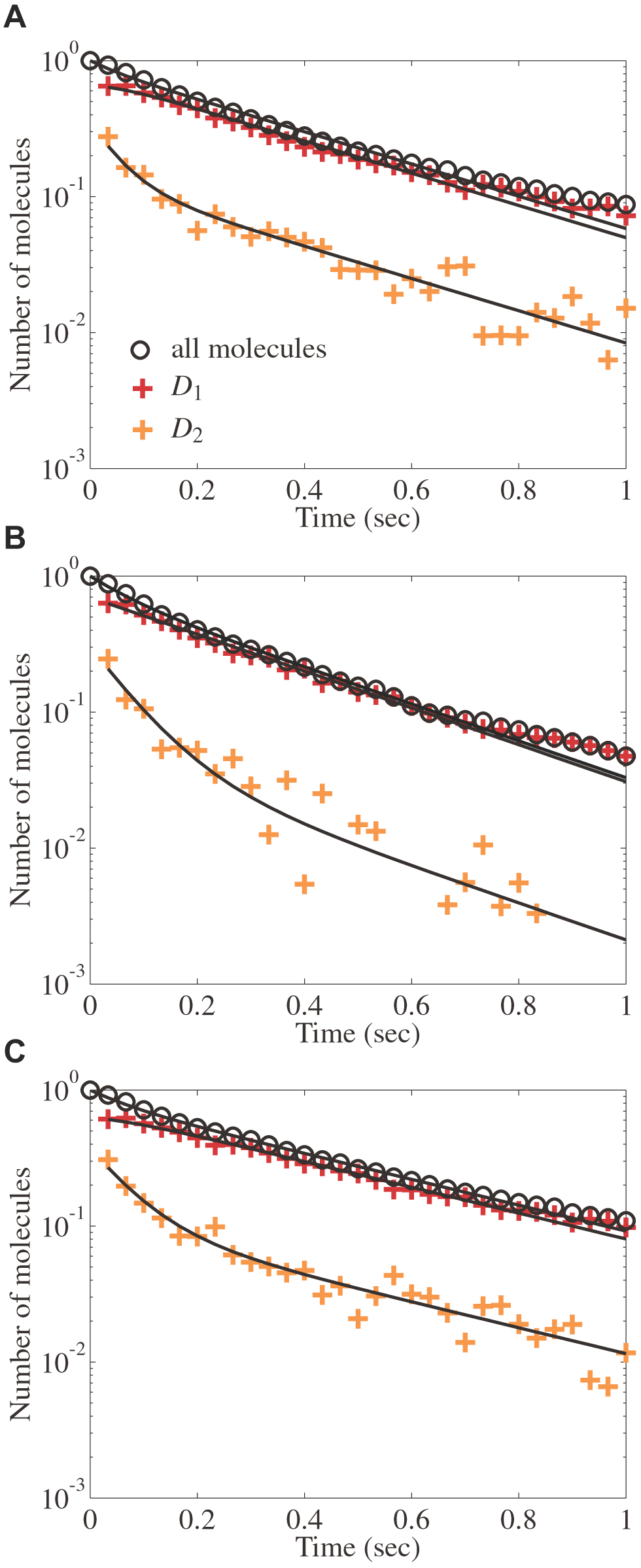

Supplement: Figure S6 — Lifetime-diffusion analysis of PTENG129E assuming two states. The dissociation curve of all molecules (open circles) and decay profiles of two subpopulations (crosses) fitted to Eqs. 8 and 9, respectively (solid lines). (A) Non-polarized cells. (B) Pseudopod. (C) Tail. (TIF) [file pcbi.1002862.s006.tif]

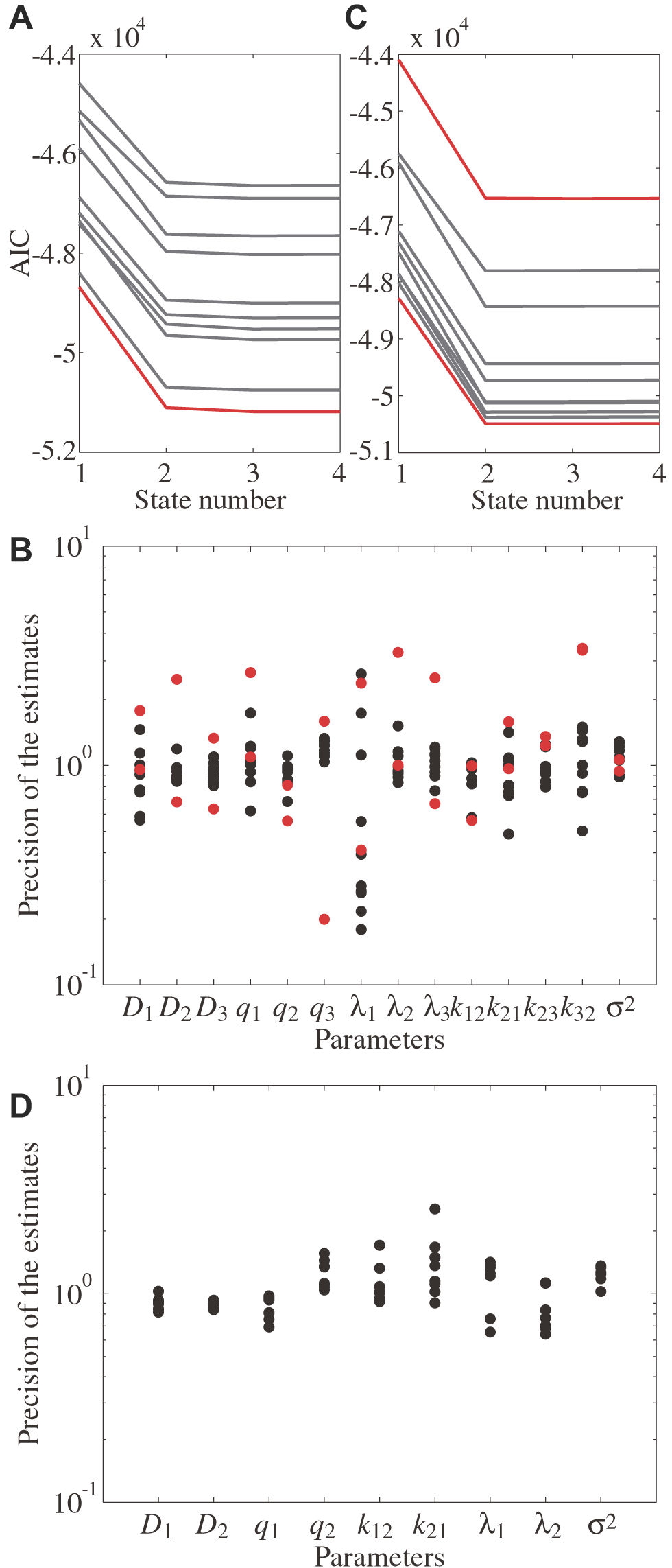

Supplement: Figure S7 — Discrimination between the two- and three-state models. 2000 trajectories were generated by numerical simulation assuming a three-state (A, B) or two-state model (C, D) for PTENG129E in non-polarized cells and analyzed. (A, C) AIC values calculated assuming 1 to 4 states with different diffusion coefficients. The results shown in red indicate wrong estimation of the state number. (B, D) The estimated parameter values of 10 independent trials of the simulation and analysis were plotted as relative values to the actual parameter values used in the simulation (Tables 1 and 2). The results shown in red in (B) indicate the estimates from the simulation assuming state 2 (see Text S1). (TIF) [file pcbi.1002862.s007.tif]
