# Supplementary material for: Asymmetric PTEN Distribution Regulated by Spatial Heterogeneity in Membrane-Binding State Transitions
Source: PLoS Comput Biol. 2013 Jan 10;9(1):e1002862. doi: 10.1371/journal.pcbi.1002862 (PMC3542079; doi:10.1371/journal.pcbi.1002862)
Supplement: Text S1 — Supporting information. (DOC) [file pcbi.1002862.s010.doc]

**SUPPORTING INFORMATION**

**Asymmetric PTEN Distribution Regulated by Spatial Heterogeneity in Membrane-Binding State Transitions**

Satomi Matsuoka, Tatsuo Shibata, Masahiro Ueda*

*Correspondence: [ueda@bio.sci.osaka-u.ac.jp](mailto:ueda@bio.sci.osaka-u.ac.jp)

TABLE OF CONTENTS

**I. Method of Lifetime-Diffusion Analysis**

Theoretical functions in Models S1 to S3…………………………………………...2

State number estimation by Akaike Information Criterion (AIC) analysis……….…4

Parameter estimation by lifetime-diffusion analysis………………………………...5

Precision of the estimates by lifetime-diffusion analysis…………………………....6

**II. Lifetime-Diffusion Analysis of Single PTEN Molecules**

Proof of imaging single PTEN_G129E_ molecules……………………………...……….9

Theoretical functions in the model for PTEN_G129E_……………………………..……9

Parameter sensitivity of the PTEN_G129E_ three-state model………………….……...11

Simulation of the molecular number based on the estimated model……………...12

Discrimination between the two-state and three-state models for PTEN_G129E_ molecules……………………………………………….………………………..…15

**III. References**…………………………………………….………………………...…17

**I. Method of Lifetime-Diffusion Analysis**

**Theoretical functions in Models S1 to S3**

*Model S1*

By solving the diffusion equation (Eq. 1) when assuming the molecule is located at the origin at time 0, the one-dimensional probability density function (PDF) of the position is written as,

$P(x,t)=\frac{e^{-\lambda t}}{\sqrt{4\pi Dt}}e^{-\frac{x^{2}}{4Dt}}.$ Eq. S1

In single-molecule imaging, the position of the molecule is estimated by fitting the fluorescence intensity profile to a two-dimensional Gaussian function. The empirical position, (*x*’(*t*), *y*’(*t*)), is the distance between the estimated positions at *t* and *t*=0. The measurement leads to an error, however, that can be incorporated into the PDF as,

$P(x',t)=\frac{e^{-\lambda't}}{\sqrt{4\pi Dt+4\pi\varepsilon^{2}}}e^{-\frac{x^{2}}{4Dt+4\varepsilon^{2}}},$ Eq. S2

where *ε* is the standard deviation (SD) of the error. *λ*’ indicates an apparent rate constant measured from the experimental data and is a summation of the actual rate constant and photobleaching rate constant such that *λ*’ = *λ*+*λ*_b_.

*Model S2*

In the absence of a state transition, the PDF of the molecular position is written as a sum of two PDFs from Eq. S2,

$P(x',t)=q_{1}\frac{e^{-\lambda_{1}'t}}{\sqrt{4\pi D_{1}t+4\pi\varepsilon^{2}}}e^{-\frac{x^{2}}{4D_{1}t+4\varepsilon^{2}}}+q_{2}\frac{e^{-\lambda_{2}'t}}{\sqrt{4\pi D_{2}t+4\pi\varepsilon^{2}}}e^{-\frac{x^{2}}{4D_{2}t+4\varepsilon^{2}}}.$ Eq. S3

Each PDF describes the molecules in states 1 and 2 as the ratios of *q*_1_ and *q*_2_, respectively, at the initial moment of membrane association (Fig. 2D).

*Model S3*

The PDF of the diffusion equations (Eq. 7) is given by the inverse Fourier transformation of *p*(*k_x_*,*k_y_*,*t*) with wave numbers, *k_x_* and *k_y_*,

$\begin{matrix} p(k_{x},k_{y},t)=\left[\left\{\left(k_{x}^{2}+k_{_{y}}^{2}\right)\left(D_{1}-D_{2}\right)+\lambda_{1}'-\lambda_{2}'\right\}\left(1-2q_{1}\right)+k_{12}+k_{21}+b\right]e^{\frac{a+b}{2}t}/4b\pi\\ -\left[\left\{\left(k_{x}^{2}+k_{_{y}}^{2}\right)\left(D_{1}-D_{2}\right)+\lambda_{1}'-\lambda_{2}'\right\}\left(1-2q_{1}\right)+k_{12}+k_{21}-b\right]e^{\frac{a-b}{2}t}/4b\pi, \end{matrix}$ Eq. S4

where

$\begin{matrix} a=-\left\{\left(k_{x}^{2}+k_{y}^{2}\right)\left(D_{1}+D_{2}\right)+k_{12}+k_{21}+\lambda_{1}'+\lambda_{2}'\right\}, \\ b=\sqrt{\left\{\left(k_{x}^{2}+k_{y}^{2}\right)\left(D_{1}+D_{2}\right)+k_{12}+k_{21}+\lambda_{1}'+\lambda_{2}'\right\}^{2}-4\left[\left\{\left(k_{x}^{2}+k_{y}^{2}\right)D_{1}+k_{12}+\lambda_{1}'\right\}\left\{\left(k_{x}^{2}+k_{y}^{2}\right)D_{2}+k_{21}+\lambda_{2}'\right\}-k_{12}k_{21}\right]}. \end{matrix}$

An inverse Fourier transformation is performed by numerical integration to obtain the PDF (Fig. 2F). The membrane residence probability, *R*(*t*), is written as,

$R(t)=(0.5+C)e^{-s_{1}t}+(0.5-C)e^{-s_{2}t}$, Eq. 8 (S5)

where

$\begin{matrix} s_{1}=-(A+B)/2, \\ s_{2}=-(A-B)/2, \\ A=-\left(k_{12}+k_{21}+\lambda_{1}'+\lambda_{2}'\right), \\ B=\sqrt{A^{2}-4\left(k{}_{12}\lambda_{2}'+k_{21}\lambda_{1}'+\lambda_{1}'\lambda_{2}'\right)}, \\ C=\frac{(k_{12}+k_{21}-\lambda_{1}'+\lambda_{2}')q_{1}+(k_{12}+k_{21}+\lambda_{1}'-\lambda_{2}')q_{2}}{2\sqrt{(k_{12}+k_{21}+\lambda_{1}'+\lambda_{2}')^{2}-4(k{}_{12}\lambda_{2}'+k_{21}\lambda_{1}'+\lambda_{1}'\lambda_{2}')}}. \end{matrix}$

The subpopulation probabilities, *Q*_1_(*t*) and *Q*_2_(*t*), are written respectively as,

$\begin{matrix} Q_{1}(t)=C_{1}e^{-s_{1}t}+C_{2}e^{-s_{2}t}, \\ Q_{2}(t)=C_{3}e^{-s_{1}t}+C_{4}e^{-s_{2}t}, \end{matrix}$ Eq. 9 (S6)

where

$\begin{matrix} C_{1}=\left\{2k_{21}-q_{1}\left(k{}_{12}+k_{21}+\lambda_{1}'-\lambda_{2}'-B\right)\right\}/2B, \\ C_{2}=\left\{-2k_{21}+q_{1}\left(k{}_{12}+k_{21}+\lambda_{1}'-\lambda_{2}'+B\right)\right\}/2B, \\ C_{3}=\left\{2k_{12}+q_{2}\left(-k{}_{12}-k_{21}+\lambda_{1}'-\lambda_{2}'+B\right)\right\}/2B, \\ C_{4}=\left\{-2k_{12}-q_{2}\left(-k{}_{12}-k_{21}+\lambda_{1}'-\lambda_{2}'-B\right)\right\}/2B. \end{matrix}$

**State number estimation by Akaike Information Criterion (AIC) analysis**

The minimum state number necessary for describing heterogeneity in the diffusion mobility was estimated by Akaike Information Criterion (AIC). When Δ*t* is sufficiently short, the displacement, Δ*r,* obtained from a mixture of diffusing molecules with various diffusion coefficients follows the PDF as described in Eq. 11. In maximum likelihood estimation (MLE), how well the distribution made from the displacement data containing *n* values fits the PDF is indicated by a log likelihood calculated for each model with different state number, *i*, as,

$l_{i}(\tilde{\theta}_{i})=\sum_{m=1}^{n} \log P_{i}(\Delta r_{m}|\theta_{i}).$ Eq. S7

MLE provides estimated values of parameters, $\tilde{\theta}_{i}$, including diffusion coefficients and their proportions by maximizing the log likelihood. When the model with a larger state number is used, it naturally results in a better fit to the data and an increase in the log likelihood. However, it simultaneously causes a complication in the model. The essential parameter number is suggested by AIC. *AIC_i_* calculated for a model with *i* states is given as,

$AIC_{i}=-2l_{i}(\tilde{\theta}_{i})+k_{i}\log(\log(n))$, Eq. S8

where *k_i_* denotes the number of parameters in the model. The second term in the right-hand side is the penalty for increasing the state number, as suggested for a Gaussian mixture model [1, 2]. The model that provides the minimal *AIC_i_* value is the one with the highest probability.

*AIC_i_* is dependent on the data size. In Eq. S8, the first term on the right hand side decreases as the data number increases, since it consists of a summation of a loss function, $-\log P_{i}(\Delta r_{m}|\tilde{\theta}_{i})$, calculated from each displacement data, Δ*r_m_*. Although the second term increases as the data number increases, the extent of the increase is generally much smaller than the decrease of the first term. When the difference between *AIC_i_* and *AIC_i_*_+1_ is taken, the relative difference is written as,

Eq. S9

and is referred to as *ΔAIC_i_* hereafter. A positive *ΔAIC_i_* value suggests that model *i* is better than model *i*+1. By increasing the number of displacement data, *ΔAIC* approximates to a constant,

,

where $E\left[-\log P_{i}(\Delta r_{m}|\theta_{i})\right]$ is the expected value of the loss function.

**Parameter estimation by lifetime-diffusion analysis**

The process of lifetime-diffusion analysis was shown in the case that a molecule adopts Models S1 or S2 by analyzing the stochastic trajectories of single molecules that were generated by numerical simulation.

*Model S1*

1000 trajectories generated by a numerical simulation using *D* = 0.01 μm^2^/s and *λ* = 1 s^-1^ were analyzed. The dissociation curve showed a single exponential decay, indicating the molecules exhibit membrane dissociation (Figure S2B). Molecular displacement during a 33 msec window was measured from the trajectories, which is shown as a histogram in Figure S2A. By fitting the mean square displacement calculated from the trajectories, the SD of the error was estimated to be 40 nm. Using the displacement data and the SD value, MLE was performed using models with 1 to 4 states. A minimum AIC value was obtained in the case of a single state (Figure S2A, inset). These results indicate that Model S1 is the most likely. The diffusion coefficient was estimated to be 0.009 μm^2^/s. By fitting the dissociation curve to Eq. 3, the dissociation rate constant was estimated to be 0.961 s^-1^. These estimated values were confirmed by introducing them into the PDF (Eq. 2), which well fitted the molecular position distribution obtained from the trajectories (Figure S2C).

*Model S2*

1000 trajectories generated by numerical simulation using *D*_1_ = 0.01 μm^2^/s, *D*_2_ = 0.10 μm^2^/s, *λ*_1_ = 0.1 s^-1^, *λ* _2_ = 1.0 s^-1^ and *q*_1_ = 0.2 were analyzed. The release curve showed an exponential decay with at least two rate constants (Figure S2E). The SD of the error was estimated to be 40 nm, and the minimum AIC value was obtained assuming that the molecule contains two states with different *D* (Figure S2D). The two diffusion coefficients were estimated to be *D*_1_ = 0.012 μm^2^/s and *D*_2_ = 0.105 μm^2^/s. The decay profiles at a longer time scale showed different exponential decays, which is an indication that the molecule adopts Model S2. Fitting these curves to Eq. 6, the dissociation rate constants were estimated to be *λ*_1_ = 0.144 s^-1^, *λ* _2_ = 0.970 s^-1^, and *q*_1_ = 0.203 (Figure S2E). The PDF from Model 5 (Eq. S3) with estimated parameter values well fitted the experimental position distribution, showing successful estimation (Figure S2F).

**Precision of the estimates by lifetime-diffusion analysis**

We evaluated the probability of a correct estimation based on the results of 10 independent analyses of simulated trajectories. For Models S1 to S3, all 10 trials ended successfully, each providing estimated values that were similar to the actual ones used in the simulation (Figure S3). The range of the deviations from the actual values was dependent on the number of trajectories analyzed. The analysis of 1000 trajectories could provide an order of magnitude estimate from the actual value. Increasing the trajectory number helped reveal the kinetics of the state transitions and membrane dissociations with further accuracy. Consider, for example, the case where the transition occurs between two states with different diffusion coefficients and same or similar dissociation rate constants. Using 1000 trajectories, the rate constant of a relatively slow process was sometimes significantly underestimated, suggesting that the analysis failed to detect the relatively rare reaction (Figures S4A and S4B). In addition, 3 of the 10 trials could not correctly discriminate between faster and slower dissociating states that had rate constants differing by 10%. Both these problems were improved by increasing the trajectory number to 3000 (Figure S4C). Since the larger data number reduces fluctuations in the decay profiles, it effectively improves the kinetics analysis so as to accurately estimate both of the similar rate constants.

The estimation accuracy was also sensitive to the quality of the single-molecule imaging data as well as its quantity. Reducing the measurement error by using higher signal-to-noise-ratio images could help in estimating both diffusion coefficients and kinetic parameters, especially when the diffusion coefficients were similar. We examined the probability of a correct estimation by analyzing 1000 trajectories generated by numerical simulation. In the simulation, Model S3 was used without changing the kinetic parameter values from those in Figures 2 and S4. When the difference between two diffusion coefficients was 5-fold and the SD of the measurement error was 40 nm, all 10 trials provided estimates similar to the actual values when *D*_1_ and *D*_2_ were set to 0.02 and 0.1 μm^2^/sec, respectively (Figure S4D). However, 2 of the 10 trials underestimated *q*_1_ when *D*_1_ and *D*_2_ were set to 0.01 and 0.05 μm^2^/sec, respectively (Figure S4E). In the latter case, when the simulation was performed using 35 nm as the SD of the error instead of 40 nm, all trials ended successfully (Figure S4F). Similar results were obtained when the difference between *D*_1_ and *D*_2_ was 2-fold, and the error reduction was also effective to increase estimation accuracy (Figure S4G-I). The underestimation is due to fluctuations in the decay profiles, and the primary cause is inadequate precision when estimating diffusion coefficients and the subpopulation ratio rather than a lack of data. Both the diffusion coefficients and the subpopulation ratio are estimated by fitting the displacement distribution to the PDF (Eq. 11, which includes the error term). The proportion of the error in the denominator on the right side of Eq. 11 is required to be sufficiently small to correctly estimate relatively small diffusion coefficients. In single-molecule imaging, the error can be decreased by increasing the excitation intensity or the exposure time duration. Lifetime-diffusion analysis primarily utilizes differences in diffusion mobilities to discriminate the molecular states and is not intended to analyze transitions between two states of equal diffusion coefficients.

**II. Lifetime-Diffusion Analysis of Single PTEN Molecules**

**Proof of imaging single PTEN_G129E_ molecules**

Visualized spots of tetramethylrhodamine (TMR) fluorescence were confirmed to be single PTEN_G129E_ molecules as follows. The process of photo-bleaching was examined by measuring the intensity of the fluorescent spots on the membrane of PTEN_G129E_-Halo expressing cells in which HaloTag proteins were labeled with TMR-conjugated HaloTag ligand and immobilized by fixation using 3.7% formaldehyde (Matsuoka et al., 2006). Under the same excitation conditions as living cells, the fluorescence intensity decreased stepwise as the fluorescent spot disappeared (Figure 3B, inset). The intensity before the photobleach followed a Gaussian distribution with a single peak, suggesting the fluorescence was derived from a single fluorophore. Fluorescent spots in the images were typically 0.4 μm in diameter, which is as theoretically expected (Matsuoka et al., 2008). In living cells, the fluorescent spots showed the same intensity and size as those observed in fixed cells (Figure 1B). These results support our conclusion that individual fluorescent spots represent single PTEN_G129E_-TMR molecules in living *Dictyostelium* cells. Furthermore, the intensity distribution of these molecules showed no significant cell-to-cell differences, suggesting that single molecules in these cells were visualized under constant excitation conditions including a constant distance between the membrane and the glass surface and a constant photobleaching rate (Figure S1A).

**Theoretical functions in the model for PTEN_G129E_**

The diffusion equations (Eq. 12) were solved after taking the Fourier transform:

$p(k_{x},k_{y},t)=c_{1}e^{\gamma_{1}t}+c_{2}e^{\gamma_{2}t}+c_{3}e^{\gamma_{3}t},$ Eq. S10

where

$$\begin{matrix} c_{1}=\frac{\left\{q_{1}\gamma_{2}\gamma_{3}+x_{1}\left(\gamma_{2}+\gamma_{3}\right)+x_{2}\right\}\left(\gamma_{1}^{2}+x_{3}\gamma_{1}+x_{4}\right)}{k_{21}k_{32}\left(\gamma_{1}-\gamma_{2}\right)\left(\gamma_{1}-\gamma_{3}\right)}, \\ c_{2}=\frac{-\left\{q_{1}\gamma_{1}\gamma_{3}+x_{1}\left(\gamma_{1}+\gamma_{3}\right)+x_{2}\right\}\left(\gamma_{2}^{2}+x_{3}\gamma_{2}+x_{4}\right)}{k_{21}k_{32}\left(\gamma_{1}-\gamma_{2}\right)\left(\gamma_{2}-\gamma_{3}\right)}, \\ c_{3}=\frac{\left\{q_{1}\gamma_{1}\gamma_{2}+x_{1}\left(\gamma_{1}+\gamma_{2}\right)+x_{2}\right\}\left(\gamma_{3}^{2}+x_{3}\gamma_{3}+x_{4}\right)}{k_{21}k_{32}\left(\gamma_{1}-\gamma_{3}\right)\left(\gamma_{2}-\gamma_{3}\right)}, \\ x_{1}=q_{1}y_{1}-q_{2}k_{21}, \\ x_{2}=q_{1}\left(y_{1}^{2}+k_{12}k_{21}\right)-q_{2}k_{21}\left(y_{1}+y_{2}\right)+q_{3}k_{21}k_{32}, \\ x_{3}=y_{1}+y_{2}+k_{32}, \\ x_{4}=y_{1}\left(y_{2}+k_{32}\right)-k_{21}\left(k_{12}-k_{32}\right), \\ y_{1}=\left(k_{x}^{2}+k_{y}^{2}\right)D_{1}+k_{12}+\lambda_{1}', \\ y_{2}=\left(k_{x}^{2}+k_{y}^{2}\right)D_{2}+k_{21}+k_{23}+\lambda_{2}', \\ y_{3}=\left(k_{x}^{2}+k_{y}^{2}\right)D_{3}+k_{32}+\lambda_{3}', \end{matrix}$$

and *γ*_1_, *γ*_2_ and *γ*_3_ are the solutions of the equation,

$\gamma^{3}+\left(y_{1}+y_{2}+y_{3}\right)\gamma^{2}+\left(y_{1}y_{2}+y_{2}y_{3}+y_{3}y_{1}-k_{12}k_{21}-k_{23}k_{32}\right)\gamma+\left(y_{1}y_{2}y_{3}-k_{12}k_{21}y_{3}-k_{23}k_{32}y_{1}\right)=0.$The PDF of the molecular position was obtained by a numerically integrated inverse Fourier transform (Fig. 4D). The membrane residence probability, *R*(*t*), is written as,

$R(t)=C_{1}e^{\gamma_{1}t}+C_{2}e^{\gamma_{2}t}+C_{3}e^{\gamma_{3}t},$ Eq. S11

where

$$\begin{matrix} C_{1}=\frac{\left\{q_{1}\gamma_{2}\gamma_{3}+X_{1}\left(\gamma_{2}+\gamma_{3}\right)+X_{2}\right\}\left(\gamma_{1}^{2}+X_{3}\gamma_{1}+X_{4}\right)}{k_{21}k_{32}\left(\gamma_{1}-\gamma_{2}\right)\left(\gamma_{1}-\gamma_{3}\right)}, \\ C_{2}=\frac{-\left\{q_{1}\gamma_{1}\gamma_{3}+X_{1}\left(\gamma_{1}+\gamma_{3}\right)+X_{2}\right\}\left(\gamma_{2}^{2}+X_{3}\gamma_{2}+X_{4}\right)}{k_{21}k_{32}\left(\gamma_{1}-\gamma_{2}\right)\left(\gamma_{2}-\gamma_{3}\right)}, \\ C_{3}=\frac{\left\{q_{1}\gamma_{1}\gamma_{2}+X_{1}\left(\gamma_{1}+\gamma_{2}\right)+X_{2}\right\}\left(\gamma_{3}^{2}+X_{3}\gamma_{3}+X_{4}\right)}{k_{21}k_{32}\left(\gamma_{1}-\gamma_{3}\right)\left(\gamma_{2}-\gamma_{3}\right)}, \\ X_{1}=q_{1}Y_{1}-q_{2}k_{21}, \\ X_{2}=q_{1}\left(Y_{1}^{2}+k_{12}k_{21}\right)-q_{2}k_{21}\left(Y_{1}+Y_{2}\right)+q_{3}k_{21}k_{32}, \\ X_{3}=Y_{1}+Y_{2}+k_{32}, \\ X_{4}=Y_{1}\left(Y_{2}+k_{32}\right)-k_{21}\left(k_{12}-k_{32}\right), \\ Y_{1}=k_{12}+\lambda_{1}', \\ Y_{2}=k_{21}+k_{23}+\lambda_{2}', \\ Y_{3}=k_{32}+\lambda_{3}'. \end{matrix}$$

Each subpopulation decays in accordance with the subpopulation probabilities:

$\begin{matrix} Q_{1}\left(t\right)=C_{11}e^{\gamma_{1}t}+C_{12}e^{\gamma_{2}t}+C_{13}e^{\gamma_{3}t}, \\ Q_{2}\left(t\right)=\left\{\left(Y_{1}+\gamma_{1}\right)C_{11}e^{\gamma_{1}t}+\left(Y_{1}+\gamma_{2}\right)C_{12}e^{\gamma_{2}t}+\left(Y_{1}+\gamma_{3}\right)C_{13}e^{\gamma_{3}t}\right\}/k_{21}, \\ Q_{3}\left(t\right)=\left\{\gamma_{1}^{2}+\left(Y_{1}+Y_{2}\right)\gamma_{1}+\left(Y_{1}Y_{2}-k_{12}k_{21}\right)\right\}C_{11}e^{\gamma_{1}t}/k_{21}k_{32} \\ +\left\{\gamma_{2}^{2}+\left(Y_{1}+Y_{2}\right)\gamma_{2}+\left(Y_{1}Y_{2}-k_{12}k_{21}\right)\right\}C_{12}e^{\gamma_{2}t}/k_{21}k_{32} \\ +\left\{\gamma_{3}^{2}+\left(Y_{1}+Y_{2}\right)\gamma_{3}+\left(Y_{1}Y_{2}-k_{12}k_{21}\right)\right\}C_{13}e^{\gamma_{3}t}/k_{21}k_{32}, \end{matrix}$ Eq. S12

where

$$\begin{matrix} C_{11}=\frac{\left\{q_{1}\gamma_{2}\gamma_{3}+X_{1}\left(\gamma_{2}+\gamma_{3}\right)+X_{2}\right\}}{\left(\gamma_{1}-\gamma_{2}\right)\left(\gamma_{1}-\gamma_{3}\right)}, \\ C_{12}=\frac{-\left\{q_{1}\gamma_{1}\gamma_{3}+X_{1}\left(\gamma_{1}+\gamma_{3}\right)+X_{2}\right\}}{\left(\gamma_{1}-\gamma_{2}\right)\left(\gamma_{2}-\gamma_{3}\right)}, \\ C_{13}=\frac{\left\{q_{1}\gamma_{1}\gamma_{2}+X_{1}\left(\gamma_{1}+\gamma_{2}\right)+X_{2}\right\}}{\left(\gamma_{1}-\gamma_{3}\right)\left(\gamma_{2}-\gamma_{3}\right)}. \end{matrix}$$

The number of rate constants in *R*(*t*) and *Q*_1-3_(*t*) is three, which is determined by the state number. The three decay profiles exhibit the same decay rate after a sufficiently long time due to the state transitions between states 1 and 2 and states 2 and 3 reaching a constant ratio of the subpopulation (Figure 4A and 4C).

**Parameter sensitivity of the PTEN_G129E_ three-state model**

In the three-state model of PTEN_G129E_ molecules in Figure 4E, parameter sensitivity was evaluated by applying up to 10% changes to each parameter value and examining the resulting deviations from the original dissociation curve and decay profiles. We learned that the dissociation curve is dependent especially on the parameters *λ*_2_, *k*_12_ and *k*_21_ (Figure S5). *λ*_2_ has the largest effect on the decay rate at the steady state with respect to the state transitions (Figure S6B). Compared to *λ*_2_, *λ*_1_ and *λ*_3_ have less effect on the overall dissociation curve. The value of *λ*_1_ is relatively small among model parameters, so that its changes have minimal effect on the decay kinetics of any state (Figure S6A). On the other hand, changes in *λ*_3_ specifically affect the decay profile of state 3. However, the molecules adopting state 3 comprise only a minority of the ensemble, and therefore have little effect on the whole system (Figure S6C). The insensitivity of the decay profiles of states 1 and 2 to changes in *λ*_3_ is due to a separation in the transition time scale: the transitions between states 1 and 2 occur much more quickly relative to those between states 2 and 3. Thus, the membrane dissociation curve of PTEN_G129E_ is largely dependent on the decay kinetics of states 1 and 2. Changes in *k*_12_ and *k*_21_ modulate the overall kinetics by primarily altering the decay kinetics of state 1. By increasing *k*_12_ or decreasing *k*_21_, the decay of state 1 is accelerated (Figure S6D and E). Increasing *k*_12_ also raises the probability of state 2 soon before 500 msec, but lowers it after this mark due to the faster decay of state 1. The same is true for the decrease in *k*_21_. *k*_23_ and *k*_32_ are relatively small, and the decay profiles of states 1 and 2 are insensitive to these changes (Figure S6F and G). The initial probabilities, *q*_1_, *q*_2_ and *q*_3_, do not affect the dissociation rates at the steady state with respect to the state transitions (Figures S6H and I). The overall kinetics are robust to the changes in *q*_2_ when the ratio between *q*_1_ and *q*_3_ is constant. Decreasing the ratio *q*_1_/*q*_3_ lowers the overall probability of membrane associations. In conclusion, the membrane binding kinetics derived from the present model can be modulated by the parameters *λ*_2_, *k*_12_, *k*_21_ and *q*_1_/*q*_3_ in a sensitive manner. In fact, modulating the kinetics with these parameters in *Dictyostelium discoideum* cells could generate the heterogeneity in the molecular behavior that accrues with cell polarity (Figure 5C).

**Simulation of the molecular number based on the estimated model**

Considering a single PTEN molecule in non-polarized cells, the probabilities of states 1, 2 and 3 on the membrane, *p*_1_(*t*), *p*_2_(*t*) and *p*_3_(*t*), respectively, and that of diffusing in the cytoplasm, *p*_0_(*t*), exhibit temporal changes, which obey the evolution equations,

$$\left\{\begin{matrix} \frac{dp_{1}(t)}{dt}=-(k_{12}+\lambda_{1})p_{1}(t)+k_{21}p_{2}(t)+\mu_{1}p_{0}(t), \\ \frac{dp_{2}(t)}{dt}=k_{12}p_{1}(t)-(k_{21}+k_{23}+\lambda_{2})p_{2}(t)+k_{32}p_{3}(t)+\mu_{2}p_{0}(t), \\ \frac{dp_{3}(t)}{dt}=k_{23}p_{2}(t)-(k_{32}+\lambda_{3})p_{3}(t)+\mu_{3}p_{0}(t), \\ \frac{dp_{0}(t)}{dt}=\lambda_{1}p_{1}(t)+\lambda_{2}p_{2}(t)+\lambda_{3}p_{3}(t)-\left(\mu_{1}+\mu_{2}+\mu_{3}\right)p_{0}(t). \end{matrix}\right.$$

*μ*_1_, *μ*_2_ and *μ*_3_ represent the frequencies (s^-1^) of membrane associations via states 1, 2 and 3, respectively, per single cytosolic PTEN molecule. Therefore, *μ*_1_*p*_0_(*t*), for example, means a probability flow (s^-1^) for the membrane association via state 1, although the actual membrane association rate (Ms^-1^) is dependent on the association rate constant (M^-1^s^-1^) between PTEN and a binding partner for state 1 on the membrane, the concentration of the accessible binding partner (M) and the concentration of cytosolic PTEN (M). Under steady state conditions, the sum of the probability flows of membrane association should equal that of membrane dissociation. We estimated the three membrane association frequencies in the non-polarized cells in which the intracellular distribution of PTEN reaches a steady state. Letting *C* be the number of PTEN molecules in the cytoplasm, *N*_cyto_, the total number of the molecules on the membrane, *N*_mem_, is written as,

$$N_{mem}=C\left(m_{1}\mu_{1}+m_{2}\mu_{2}+m_{3}\mu_{3}\right),$$

where

$$\begin{matrix} m_{1}=\left\{(k_{21}+k_{23}+\lambda_{2})(k_{32}+\lambda_{3})-k_{23}k_{32}+k_{12}(k_{32}+\lambda_{3})+k_{12}k_{23}\right\}/m_{4}, \\ m_{2}=\left\{k_{21}(k_{32}+\lambda_{3})+(k_{32}+\lambda_{3})(k_{12}+\lambda_{1})+k_{23}(k_{12}+\lambda_{1})\right\}/m_{4}, \\ m_{3}=\left\{k_{21}k_{32}+k_{32}(k_{12}+\lambda_{1})+(k_{21}+k_{23}+\lambda_{2})(k_{12}+\lambda_{1})-k_{12}k_{21}\right\}/m_{4}, \\ m_{4}=(k_{12}+\lambda_{1})(k_{21}+k_{23}+\lambda_{2})(k_{32}+\lambda_{3})-k_{23}k_{32}(k_{12}+\lambda_{1})-k_{12}k_{21}(k_{32}+\lambda_{3}). \end{matrix}$$

The membrane/cytoplasm ratio is *R*_mc_=*N*_mem_/*N*_cyto_=*m*_1_*μ*_1_+*m*_2_*μ*_2_+*m*_3_*μ*_3_. Since *μ*_1_, *μ*_2_ and *μ*_3_ are relative to *q*_1_, *q*_2_ and *q*_3_, respectively, *R*_mc_ can be rewritten as *μ*(*m*_1_*q*_1_+*m*_2_*q*_2_+*m*_3_*q*_3_)* ,* where *μ* is a constant which corresponds to a frequency of membrane associations via all states. Using *m*_1_, *m*_2_, *m*_3_, *q*_1_, *q*_2_, *q*_3_ and *R*_mc_, which are all obtained from the experimental data, *μ* was calculated to be 2.28 s^-1^ and provided the estimates of the apparent three association frequencies: *μ*_1_ = 0.54, *μ*_2_ = 1.38 and *μ*_3_ = 0.36 s^-1^. Note that these values depend on the concentration of the membrane binding partner.

In polarized cells, assuming that *μ* at the tail membrane shares the same constant value with that of non-polarized cells, the three association frequencies at the tail could be estimated to be *μ*_1_ = 0.68, *μ*_2_ = 1.32 and *μ*_3_ = 0.27 s^-1^. The number of molecules that began membrane association within a unit time interval in a unit area in the pseudopod was approximately 60% that at the tail, a direct result of counting single molecules in 5 cells. Thus, *μ* and the three association frequencies at the pseudopod were estimated to be *μ* = 1.43, *μ*_1_ = 0.06, *μ*_2_ = 1.21 and *μ*_3_ = 0.16 s^-1^. These values were used to simulate the effect of cell polarity. Membrane-bound molecules were divided into two subgroups: bound to the pseudopod or bound to the tail membrane. The probabilities *p*_1_(*t*), *p*_2_(*t*) and *p*_3_(*t*) were given to the subgroups. The area of the pseudopod membrane was constant at out of 5%, 10%, 15%, 20% or 25% that of the whole membrane and determined the probability that a single cytosolic molecule binds to the pseudopod when the molecule associates with the membrane. Thus, the association frequencies in the evolution equations for the individual subgroups at the pseudopod and tail were proportional to the area. Once bound to the membrane, any exchange between subgroups was inhibited and the rate constants of the state transitions and membrane dissociations were dependent on the region at which the molecule was bound. The steady state probabilities were calculated for the non-polarized cell and then used them as initial values to calculate the time evolution for the polarized cell after time 0. The probabilities reached steady state after several seconds when the ratio of the pseudopod to tail density was 1 to 2.5 independent of the area of the pseudopod membrane. This ratio is exclusively determined by the association frequencies and dissociation rates of the two subgroups coupled with the same cytosolic molecules.

**Discrimination between the two- and three-state models for PTEN_G129E_ molecules**

Based on AIC analysis, we concluded the behavior of PTEN_G129E_ molecules is best described by three states. The difference between *AIC_2_* and *AIC_3_* was confirmed by examining *ΔAIC_3_* (Eq. S9), which was calculated from all displacement data (n=29914) and equaled 0.02. AIC analysis was performed after randomly choosing 10, 100, 1000, 3000 or 10000 displacement data points from the population, finding *ΔAIC_3_* was almost constant when the data sampling reached 1000 or greater. This result suggests that our data number is sufficiently large to distinguish which model, the 2-state or 3-state, most closely resembles the displacement distribution.

To confirm the AIC analysis conclusions, we generated molecular trajectories by numerical simulation using the estimated parameter values in the three-state model of non-polarized cells and performed lifetime-diffusion analysis accordingly. A simulation of 10 independent trials and analysis of 2000 trajectories resulted in *AIC_3_* being less than *AIC_2_*. Furthermore, nine trials also showed *AIC_3_* was less than *AIC_4_*, and even the one exception showed only a slight difference (Figure S7A). Additionally, because the proportion of the 4th state in this one trial was less than 1%, it could still be assumed to have three states without compromising the analysis. In all ten trials, parameter estimation gave parameter values within an order of magnitude of the original values used in the simulation (Figure S7B). Therefore, although it is possible that the AIC analysis can overestimate the state number of a system, we feel this is unlikely for our PTEN_G129E_ model.

We confirmed that the probability that PTEN_G129E_ molecules adopt only two states is sufficiently low. 2000 trajectories were synthesized by numerical simulation using the parameter values in a two-state model and analyzed by lifetime-diffusion analysis. In eight of the ten independent trials, the state number was estimated to be two (Figure S7C) and the parameter values estimated were similar to the original values used when generating the trajectories (Figure S7D). In the two exceptions, the state number was overestimated to be three and the parameter values estimated resembled those of the three-state model (Figure S7B). The probability of the overestimation was lower than that of the correct estimation, suggesting lifetime-diffusion analysis should predict a two-state model correctly.

**III. References**

1. Hartigan JA (1985) A failure of likelihood asymptotics for normal mixtures. In: LeCam L, Olshen RA, editors. Proceedings of the Berkeley Conference in Honor of Jerzy Neyman and Jack Kiefer. Monterey: Wadsworth. Vol. II, pp. 807-810.

2. Amari S, Park H, Ozeki T (2006) Singularities affect dynamics of learning in neuromanifolds. Neural Comput*.* 18(5):1007-65.
